# Responses to High Seawater Temperatures in Zooxanthellate Octocorals

**DOI:** 10.1371/journal.pone.0054989

**Published:** 2013-02-06

**Authors:** Paul W. Sammarco, Kevin B. Strychar

**Affiliations:** 1 Louisiana Universities Marine Consortium, Chauvin, Louisiana, United States of America; 2 Annis Water Resources Institute, Grand Valley State University, Muskegon, Michigan, United States of America; University of Hull, United Kingdom

## Abstract

Increases in Sea Surface Temperatures (SSTs) as a result of global warming have caused reef-building scleractinian corals to bleach worldwide, a result of the loss of obligate endosymbiotic zooxanthellae. Since the 1980’s, bleaching severity and frequency has increased, in some cases causing mass mortality of corals. Earlier experiments have demonstrated that zooxanthellae in scleractinian corals from three families from the Great Barrier Reef, Australia (Faviidae, Poritidae, and Acroporidae) are more sensitive to heat stress than their hosts, exhibiting differential symptoms of programmed cell death – apoptosis and necrosis. Most zooxanthellar phylotypes are dying during expulsion upon release from the host. The host corals appear to be adapted or exapted to the heat increases. We attempt to determine whether this adaptation/exaptation occurs in octocorals by examining the heat-sensitivities of zooxanthellae and their host octocoral alcyonacean soft corals – *Sarcophyton ehrenbergi* (Alcyoniidae), *Sinularia lochmodes* (Alcyoniidae), and *Xenia elongata* (Xeniidae), species from two different families. The soft coral holobionts were subjected to experimental seawater temperatures of 28, 30, 32, 34, and 36°C for 48 hrs. Host and zooxanthellar cells were examined for viability, apoptosis, and necrosis (*in hospite* and expelled) using transmission electron microscopy (TEM), fluorescent microscopy (FM), and flow cytometry (FC). As experimental temperatures increased, zooxanthellae generally exhibited apoptotic and necrotic symptoms at lower temperatures than host cells and were expelled. Responses varied species-specifically. Soft coral hosts were adapted/exapted to higher seawater temperatures than their zooxanthellae. As with the scleractinians, the zooxanthellae appear to be the limiting factor for survival of the holobiont in the groups tested, in this region. These limits have now been shown to operate in six species within five families and two orders of the Cnidaria in the western Pacific. We hypothesize that this relationship may have taxonomic implications for other obligate zooxanthellate cnidarians subject to bleaching.

## Introduction

Many invertebrates possess endosymbionts that support the metabolism and other physiological activities in the host and, often, the host also provides nutrient resources to the endosymbionts. Scleractinian corals possess endosymbiotic dinoflagellates of the genus *Symbiodinium*, also known as zooxanthellae [1], [2]. These microalgae provide photosynthates comprised of carbohydrates, fatty acids, glycerol, tri-glycerids, amino acids, and oxygen to the host coral tissue. The coral host, on the other hand, provides carbon dioxide and nutrients in the form of waste products (N, P, and S) and urea to the zooxanthellae *in hospite* [3]–[6] - *i.e.,* while they are still within the host, Zooxanthellae provide 65–100% [4]–[6] of the host coral’s metabolic energy requirements, although other investigators have determined that the host corals receive a substantial portion of their metabolic requirements from plankton, organic, and inorganic matter in the water column [7]–[11]. This symbiotic relationship facilitates precipitation of the calcium carbonate skeleton and colony growth through skeletal extension [2], [3], [12]–[14].

Endosymbiotic zooxanthellae are not restricted in occurrence to scleractinian corals [15], [16] and are found in bivalves (e.g. *Tridacna gigas* [17], [18], scyphozoans (e.g., *Cassiopea xamachana*; [19], [20]), and flatworms (e.g., *Amphiscolops* sp [21]), as well as in other cnidarians, such as sea anemones [*Anthopleura ballii* [22]). One marine group in which they may be commonly found is the Octocorallia. In particular, they may be found in alcyonacean soft corals [23]. Research on *Sarcophyton, Sinularia, Xenia, Lobophytum*, and others from this group has demonstrated that a similar relationship exists between the zooxanthellae and their soft coral hosts [24], [25]. Through feeding, the coral polyp can obtain organic carbon that is used by the zooxanthellae to produce needed nutrients, to produce metabolic carbon dioxide via respiration, or to be excreted as organic carbon waste [11]. The metabolic carbon dioxide produced by corals and zooxanthellae is a source of inorganic carbon in addition to the hydrogen carbonate ions in seawater. These compounds can be precipitated as skeletal calcium carbonate through a calcification process, excreted as waste, or, through photosynthesis, used by zooxanthellae to continue the energy cycle.

The zooxanthellae facilitate the precipitation of calcium carbonate micro-spicules within the tissues of the soft corals. The symbiotic relationship in both scleractinians and octocorals generally operates within a defined temperature range of ∼18 to 33°C [26] with optimal temperatures at 25 to 29°C [27]. Exceptions include, for example, reefs in the Persian Gulf, which have adapted to temperatures ranging from 13 to 38°C [28]. Corals, when exposed to seawater temperatures above normal levels for their region, will exhibit “bleaching”; *i.e.,* they lose their zooxanthellae, which provide color to the host coral tissue, leaving the tissue transparent. This has become one of several major causes of reef decline in the world, including pollution (P and S, which can also cause bleaching), disease, and other perturbations. Thus, the colony becomes “white” due to exposure of the skeleton through unpigmented tissue. Once the zooxanthellae are lost, if another population of zooxanthellae is not re-established within the coral host tissue within a few days to a few weeks, the coral will die [29]–[32]. This is also dependent upon the coral’s environment returning to pre-stress conditions. Bleaching can be caused by other factors, such as salinity, disease, pollution, and possibly ocean acidification, but these will not be considered in this paper.

Scleractinians appeared as a taxonomic group in the mid-Triassic, approximately 240 million years before present [33]. Stanley and van de Schootbrugge [34], [35] proposed that the co-evolution of endosymbiotic zooxanthellae emerged in the late Triassic. All sub-groups within the Octocorallia appear to have emerged during the Cretaceous. The possession of the ability to accept endosymbiotic zooxanthellae or other endosymbiotic organisms is still evident today and would appear to be a highly conserved trait, having been retained for hundreds of millions of years.

It is now known that, in this symbiotic relationship, some Indo-Pacific scleractinian coral hosts are exapted or adapted to above-normal seawater temperatures – temperatures that induce bleaching and the loss of zooxanthellae from the coral. Exaptation is defined as a character that has evolved for another function, or no function at all, but which has been co-opted for a new use [36]–[42]. Recent studies indicate that the hosts have higher temperature tolerances than their zooxanthellae. It is likely that it is the photosynthetic machinery which is temperature-sensitive in the zooxanthellae [43]–[45]. In the host cnidarians, however, it is not known whether this character represents an adaptation to higher seawater temperatures which had been experienced earlier in their evolutionary history, or an adaptation to another selective factor in its evolutionary history separate from temperature but now fortuitously playing a role in temperature-tolerance (exaptation). It is not our purpose here to attempt to differentiate between these two processes; the question remains unresolved.

Earlier experimentation has demonstrated that zooxanthellae exposed to high seawater temperatures exhibit high levels of apoptosis and necrosis while *in hospite*, prior to their separation from the host [42], [46]–[50]. Apoptosis and necrosis are forms of programmed cell death (PCD), characterized by abnormal changes in cell proliferation, including dysregulation of cell development [46], [51]–[53]. Kerr et. al. [54] first described apoptosis, which is now considered to be the most prominent form of PCD. Apoptosis can be mediated by either internal or external stress and functions by regulating cytological and molecular aspects of cell differentiation, organogenesis, and body structure. Since Vogt’s [55] original description of PCD, six additional types of vertebrates and invertebrates have been found to exhibit this phenomenon. Only one type of PCD, however, - apoptosis - has thus far been documented in cnidarians [47], [48], [56], [57]. Apoptosis is recognized by chromatin condensation and DNA fragmentation within the cell and has been observed in hydrozoans (e.g. *Hydra* sp. [56]), schyphozoans (e.g. true jellyfish [58]) and some anthozoans (e.g. anemones [59]; scleractinian corals [50]).

Apoptosis does not always occur in association with bleaching, however. Ralph et al. [60] have shown that some zooxanthellae released by scleractinian corals are in fact functional at temperatures several degrees above the bleaching threshold. These results are consistent with Strychar et al. [47] who showed that although >95% of the symbiont cells lost from a host coral during a bleaching episode may be apoptotic or necrotic, some (e.g. ∼5%) remain viable for short periods of time. Hence, it is possible for symbiont cells to survive and potentially be reabsorbed for symbiosis [6]. Re-association, however, is likely time-limited, given the expelled cells are considered “naked” - dinoflagellates consisting of a cell membrane but lacking a thick cellulose cell wall [61], [62].

During exposure to the high temperatures, most of the zooxanthellae have already entered a process of cell death before being released from the host [42], [47], [63]. The high mortality levels observed in this zoothanthellar population is an indicator of their high degree of heat sensitivity leading to photodamage. The photodamage is in turn associated with damage to the D1 protein complex, resulting from reactive oxygen species (ROS) being produced within the PSII photosystem [64]–[66]. High levels of ROS are also observed during UV exposure [67]–[69] and, in addition to the D1 protein, are known to damage the carbon-fixing protein Rubisco [61], [62], [70], [71]. They also inhibit the biosynthesis of the D1 protein [72] as well as the repair of photodamage [73]. Hoogenboom et al. [74] has shown that PS1 is comparatively tolerant of high heat stress, as opposed to the PSII system and the entire chain electron transport function. In particular, these authors found that, under conditions of high heat stress, cyclic electron flow increased from PSII to PSI. It also increased light absorption of PSI at the expense of PSII. All of this appears to cause a 50% reduction in phosphoglycolate phosphatase (PGPase), the first enzyme involved in photorespiration. This, in turn, causes a subsequent decline in zooxanthellar productivity [75], which may be the ultimate underlying ‘cause’ of coral bleaching.

In general, scleractinian coral hosts may not require a high degree of adaptation to rising seawater temperature [42], compared to zooxanthellae. The coral hosts appear to be less sensitive to temperature and are already adapted (or exapted) to a high degree. This is not without variation, however. If one contrasts coral hosts within [76], [77] or between environments, this differential sensitivity between coral host and zooxanthellae may be seen to vary species-specifically. Baird et al. [78] discusses mechanisms by which the host also plays an important role in the bleaching process. He cites cases where host adaptation to the perturbations are also important. In our earlier experiments, scleractinian coral hosts did not exhibit signs of apoptosis or necrosis until exposed to experimental temperatures as high as 34°C [42], unlike their *Symbiodinium* endosymbionts. This implies that the zooxanthellae in this holobiont are doing most of the adapting to temperature in these cases.

It also appears that this relationship regarding differential sensitivity to high seawater temperatures between the host and the symbiont occurs in several taxa within the Cnidaria. This is because it has been demonstrated in three species from three different scleractinian families in the Indo-Pacific (Great Barrier Reef) – *Acropora hyacinthus* (Acroporidae), *Favites complanata* (Faviidae), and *Porites solida* (Poritidae) [46], [50]. The question arose regarding whether this response was region-specific to the western Indo-Pacific, or the Great Barrier Reef. It is now known that the same cellular responses occur under the same conditions in the Caribbean species *Montrastraea cavernosa* and *Millepora alcicornis* [79]. These authors also found that bleaching susceptibility is decreased in coral and their symbionts when species have been exposed to prior bleaching events (*i.e.* high heat stress). This may be particularly relevant to shallow water (e.g. 7–10 m) species or those living at the air-water interface, subject to aerial exposure during low tides.

In support of this concept, Cervino et al. [80] concluded that under conditions of stress, host corals synthesize more heat shock proteins than their symbionts, making the host less sensitive to heat stress. Vivekanandan and Jayasankar [81] found that, in the Indian Sea region (Gulf of Kachchh, Gulf of Mannar, Lakshadweep Sea, Nicobar Sea, and the Andaman Sea), zooxanthellae were more sensitive to stress than coral tissue. Similarly, Harithsa et al. [82] found that coral located in Kavaratti Atoll (India) were less sensitive to temperature stress than their symbionts. Though not temperature stress related, Ambariyanto and Hoegh-Guldberg [83] speculated that zooxanthellae are more sensitive to nutrient enrichment than the host. Geddes [84], and McLaughlin and Zahl [85], similarly speculated that zooxanthellae “serve the host”, and that they are more sensitive to perturbations than any of their hosts.

Endosymbiotic zooxanthellae from soft corals also undergo apoptosis and necrosis upon expulsion from their hosts, as has been demonstrated in controlled laboratory experiments [47]. Mortality due to heat stress is not to be confused with mortality of zooxanthellae due to natural aging and subsequent expulsion [86], [87]. What is not known, however, is whether the host octocoral, particularly alcyonaceans, exhibit differential heat-sensitivity to increased seawater temperatures in comparison to their *in hospite* zooxanthellae. Alcyonacean octocorals are known to bleach during periods of above-normal monthly maximum temperatures [25], [88]–[90], in a manner similar to scleractinians. Their symbionts have also been documented to exhibit apoptosis [47], [91] and experience mortality [92] and sometimes mass mortality [93], [94], during severe bleaching episodes. Soft corals are in some cases less sensitive to increased seawater temperatures than scleractinian corals, as has been observed in Sri Lanka [95]. In that case, the soft corals bleached later and to a lesser extent than scleractinians in the same area [96]. Alyconacean soft corals in South Africa have also declined significantly in abundance from 1993 to 2006 [97]. Is heat sensitivity in *in hospite* zooxanthellae higher than that of their soft coral hosts, as has been observed in scleractinians? If so, and if the limits of this physiological relationship are exceeded regularly, there could be a mass local extinction of those cnidarian populations. Also, are octocorals similarly or better-adapted to heat sensitivity than their scleractinian counterparts? In the case where one or more soft corals are highly sensitive to high seawater temperatures, they might serve well as environmental indicators of above average Mean monthly Maximum Temperatures (MMT).

Thus far, octocorals are known to harbor five clades/phylotypes of zooxanthellae - A–D [98] and G [99]. It is known that Phylotype D1 is more thermally tolerant than other clades occurring in the soft corals, such as C1. It has higher saturated PUFAs (polyunsaturated fatty acids), which enhance thermal stability by reducing susceptibility of the membrane to ROS (reactive oxygen species). Clones which are less thermally tolerant cannot modify their lipid content (which may lead to destabilization of symbiosis). It is not known what effects heat might have on the enzymes in these clades that are responsible for desaturating fatty acids [100], [101].

Different *Symbiodinium* phylotypes occur in different host species, and possess different temperature tolerances [102], [103], [104]. In addition, there is species-specific variation in host physiology in, e.g., antioxidant properties, UV-absorbing proteins, etc. These apoptotic and necrotic responses in the zooxanthellae under high heat conditions, however, have now been observed in three families of the Scleractinia in two oceans, and two families of the Octocorallia in the western Indo-Pacific. Although there may be variation between these taxa regarding observed responses, there are clearly parallel responses as well [79], [81].

Apoptosis has been observed in each of the other eight known phyla (e.g. Annelida [105]; Arthropoda [106]; Chordata [54]; Echinodermata [107]; Mollusca [108]; Nematoda [109], [110]; Porifera [111]). Up until now, it has not been known whether apoptosis occurs in octocoral host tissue.

In this study, we ask the following questions: 1) Do apoptosis and necrosis occur in the soft coral host and/or in the *in hospite* endosymbiotic zooxanthellae, in a manner similar to that observed to occur in scleractinians? 2) If so, are the zooxanthellae differentially susceptible to heat stress when compared to their octocoral hosts? 3) Does this differential susceptibility to heat stress vary from the relationship known to exist in scleractinians? 4) If so, does it vary inter-specifically?

## Results

### Experimental Temperatures

**Figure 1:**
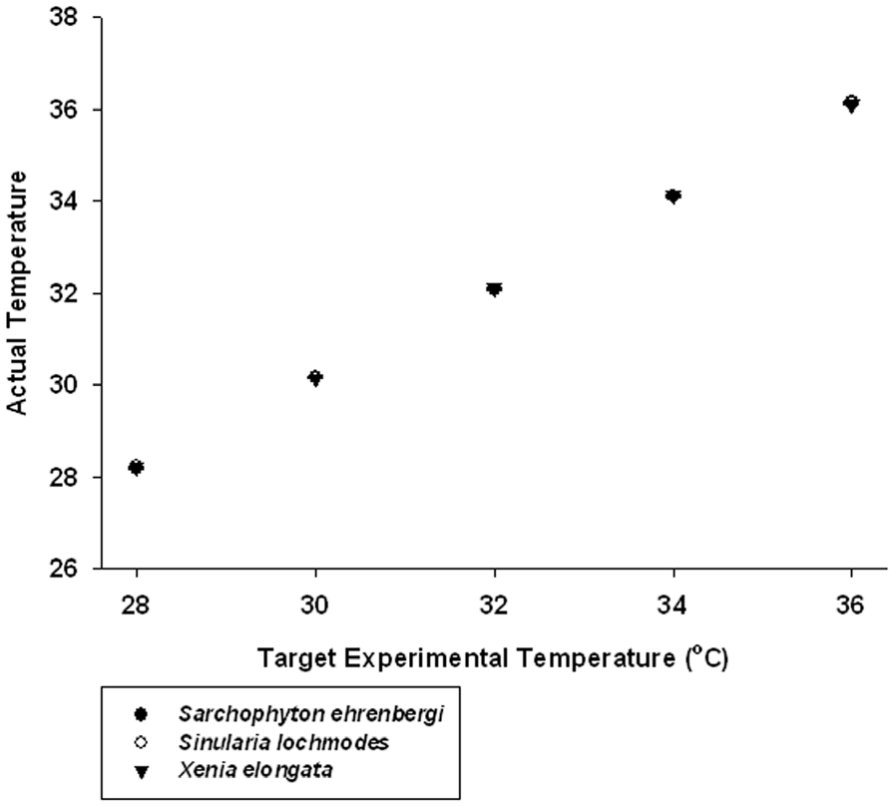
Graph of the relationship between target temperatures in this experiment (28, 30, 32, 34, and 36°C) vs. the actual measured temperature values. Means and 95% confidence limits shown. Each mean is a grand mean of 360 values measured over 10 trials (n_i_ = 36) for each temperature. Values plotted for the three alcyonacean soft coral species encompassed in this experiment: • = *Sarchophyton ehrenbergi*, ○ = *Sinularia lochomodes*, and ▾ = *Xenia elongata*. Points overlay each other. 95% confidence limits are so small that they are difficult to discern and are obscured by the points, indicating that experimental temperartures were close to target values and precise.

The monitored seawater temperatures in the tanks were very close to the target values assigned to the treatments. A review of Fig. 1 indicates that not only were these values close to their original target values, the variance around the means through all trials was so low that 95% confidence limits were difficult to discern. In addition, the Comparison of a Single Observation with the Mean of a Sample tests revealed that six of the 150 runs had significantly different temperatures than their target temperatures (p<0.05–0.001; see Table S1). These were *Sarcophyton ehrenbergi*, 30°C, runs #1 and 4; 36°C, runs #7 and 9; *Sinularia lochmodes*, 28°C, run #8; and *Xenia elongata*, 32°C, run #6. Even though these were significantly different than target, the range of differences between the mean experimental temperature and the target in these cases was 0.15 to 0.24°C. We do not believe that these minor differences in these few cases compromised the results of the experiment.

### Overall Response Patterns in Coral Host and *Symbiodinium* Cells

It should be noted that, in all 18 two-way ANOVAs, viable cell frequency, apoptotic cell frequency, and necrotic cell frequency exhibited highly significant changes (p<0.001) in response to increasing experimental temperatures. The same was true for different times of sampling; they were all highly significantly different (p<0.001). Also in all cases, however, the higher-order interaction term of temperature × time was highly significant (p<0.001); this indicates that responses of the **v**ariable in question to temperature were not comparatively uniform through time; they varied with the specific time of sampling (e.g., yielding a linear vs. a non-linear response within the same sub-experiment). This variability in response is clearly discernible in the graphs.

### Transmission Electron Microscopy (TEM)

#### Morphological changes in *symbiodinium* cells expelled from soft corals

**Figure 2:**
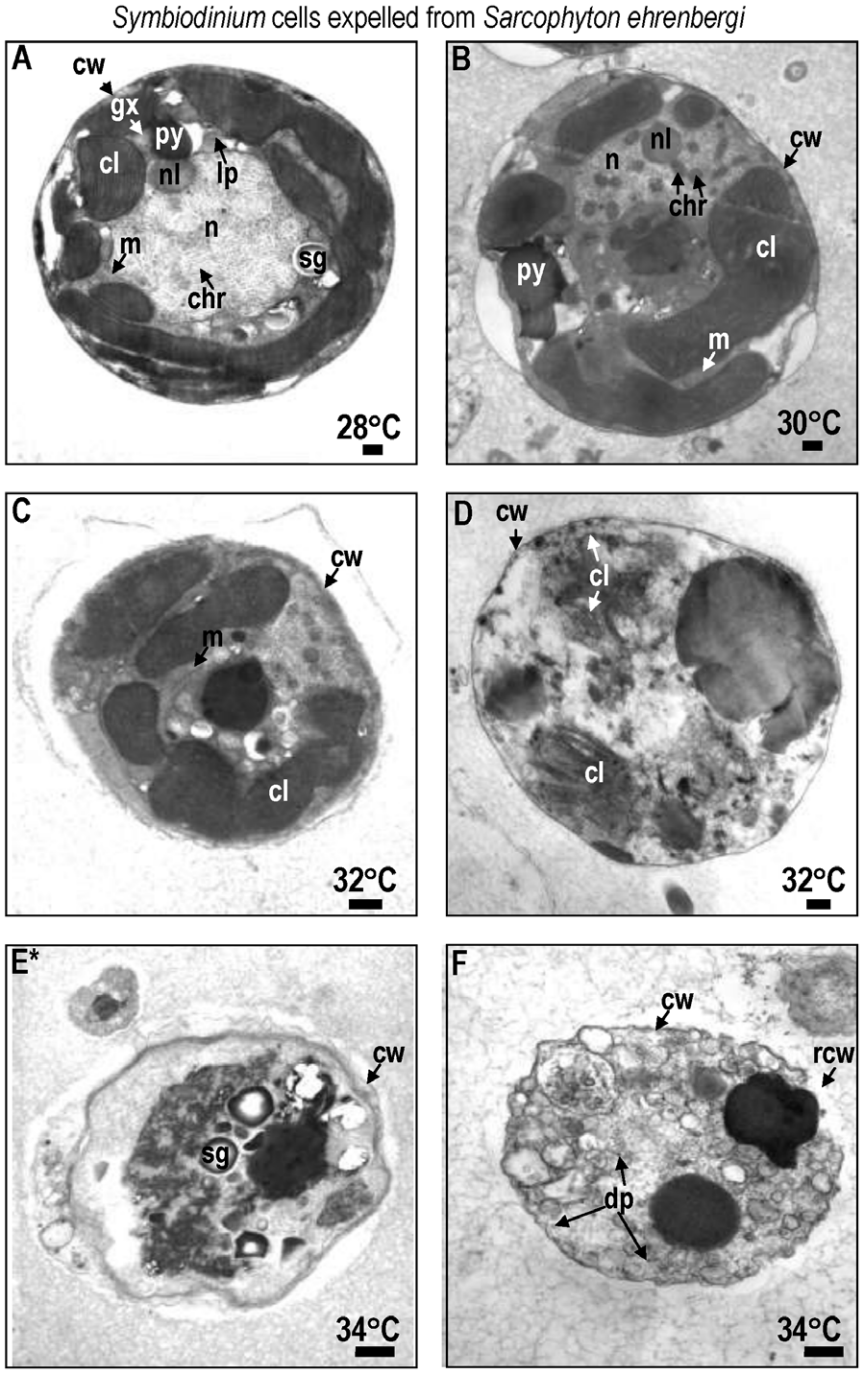
Transmission Electron Micrograph (TEM) of *Symbiodinium* cells lost from *Sarcophyton ehrenbergi* at temperatures 28 to 34°C. Most *Symbiodinium* cells appeared normal at 28 to 30°C (A,B). Symptoms of reversible early- to mid-stage apoptosis (C) or necrosis (D) occurred at 32°C within 12 h. After 9 h at 34°C, many *Symbiodinium* cells exhibited symptoms of late non-reversible apoptosis (E) and necrosis (F). Symbols: ab = apoptotic body, chr = chromosome, cl = chloroplast, cw = cell wall, gx = glyoxysome, m = mitochondria, n = nucleus, py = pyrenoid, sg = starch grain. Scale bar = 500 nm.

**Figure 3:**
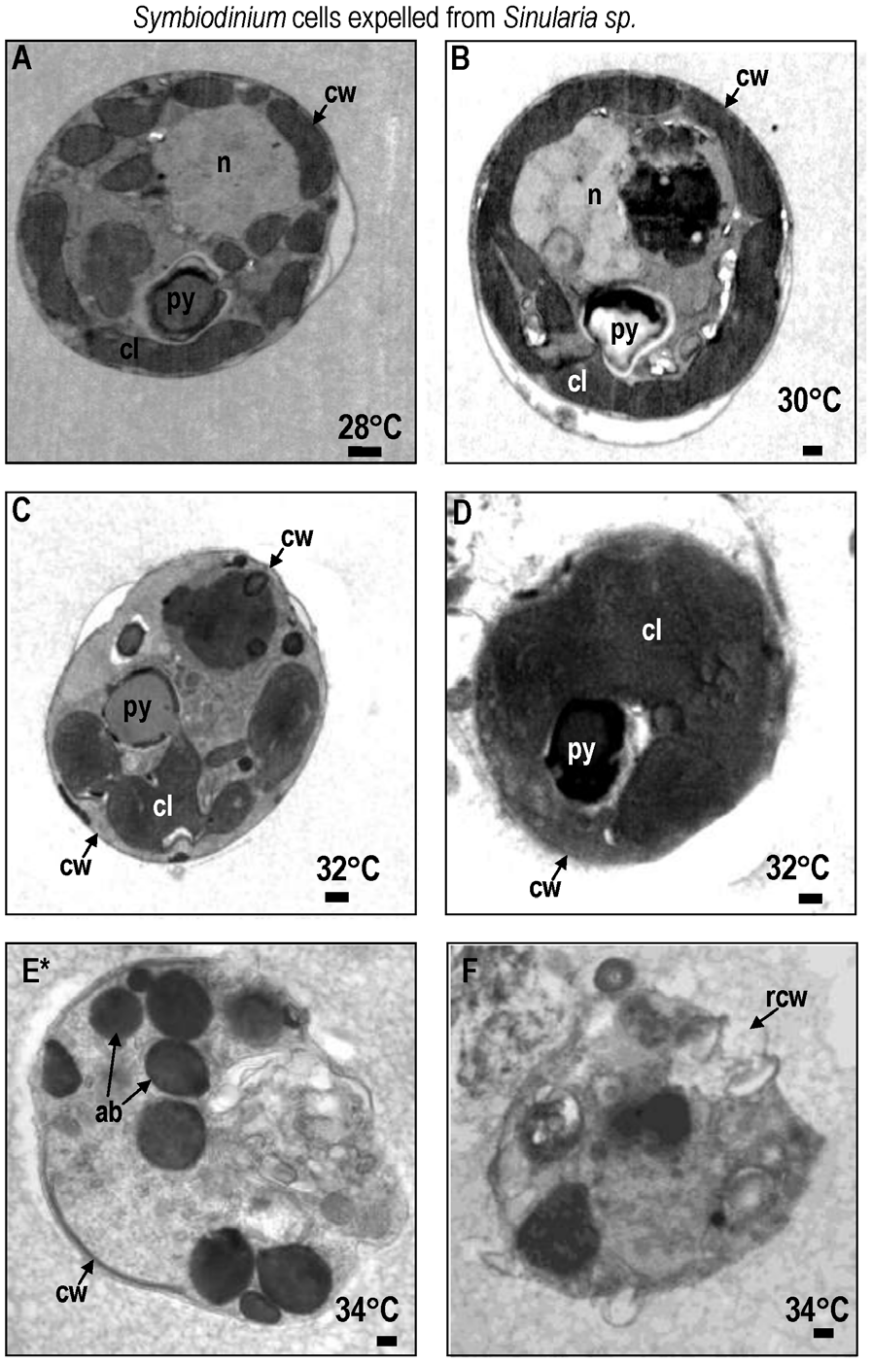
TEM of *Symbiodinium* cells lost from *Sinularia lochmodes* at temperatures 28° to 34°C. Most *Symbiodinium* cells appeared normal at 28 to 30°C (A,B). Symptoms of reversible early- to mid-stage apoptosis (C) or necrosis (D) occurred at 32°C within 12 h. After 9 h at 34°C, many *Symbiodinium* cells exhibited symptoms of late non-reversible apoptosis (E) and necrosis (F) symptoms. Symbols: ab = apoptotic body, chr = chromosome, cl = chloroplast, cw = cell wall, gx = glyoxysome, m = mitochondria, n = nucleus, py = pyrenoid, rcw = ruptured cell wall, sg = starch grain. Scale bar = 500 nm.

At 28° and 30°C, *Symbiodinium* cells exhibited normal characteristics in both *Sarcophyton ehrenbergi* (Fig. 2a,b) and *Sinularia lochmodes* (Fig. 3a,b; see [46], [112] for detailed description of normal zooxanthellar cell characteristics). Viable cells were coccoid in shape, measuring 12±1.3 µm in size (mean±95% confidence intervals; CI) and possessed multi-lobed and elongated chloroplasts with distinct lamellae. At 32° and 34°C, the chloroplasts, nuclei, and mitochondria of symbiotic zooxanthellae become fragmented and began to lose their identifying morphological characteristics (Fig. 2c,d for *Sarchophyton ehrenbergi*; Fig. 3c,d for *Sinularia lochmodes*). Apoptotic cells shrank to 6.5 µm at the higher temperatures (±2.4 µm; Fig. 2e, *Sarcophyton ehrenbergi*; Fig. 3e, *Sinularia lochmodes*). The cytoplasmic organelles, normally seen in loose aggregates, fused together forming large organelle bodies. At later stages of apoptosis, individual chloroplast organelles were no longer visible (Fig. 2e, *Sarcophyton ehrenbergi*; Fig. 3e, *Sinularia lochmodes*). Lamellae, however, were occasionally observed as free-floating throughout the cell cytoplasm (Fig. 2d, *Sarcophyton ehrenbergi*). Necrosis was also observed at elevated temperatures (Fig. 2f, *Sarcophyton ehrenbergi*; Fig. 3f, *Sinularia lochmodes*). These necrotic cellular characteristics were short in duration because the zooxanthellar cell membrane quickly disintegrated. Cytosolic contents were released into the symbiosome in both *Sarcophyton ehrenbergi* and *Sinularia lochmodes* or into the water column.

**Figure 4:**
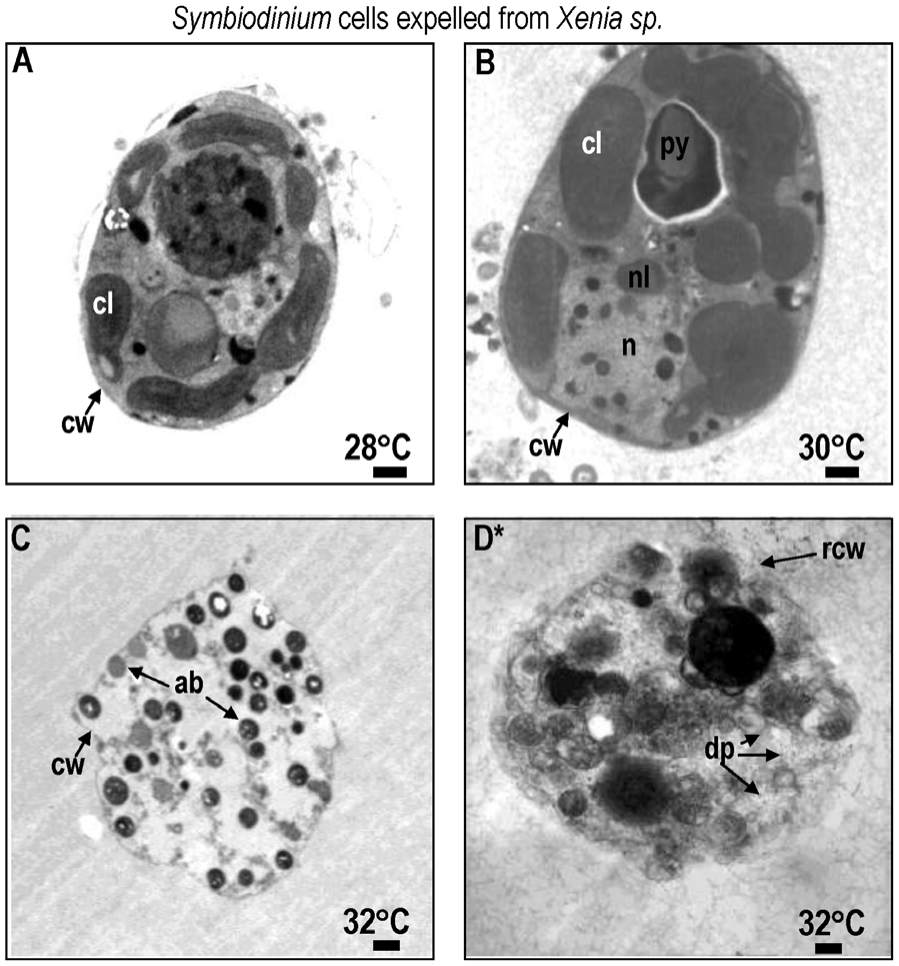
TEM of *Symbiodinium* cells lost from *Xenia elongata* at temperatures of 28 to 32°C. Most *Symbiodinium* cells appeared normal at 28°C (A). Symptoms of cell death become evident at 30°C as organelles move to opposing ends of the cell (B). Symptoms of irreversible late stage apoptosis (C) or necrosis (D) occurred at 32°C within 9 h. Symbols: ab = apoptotic body, chr = chromosome, cl = chloroplast, cw = cell wall, gx = glyoxysome, m = mitochondria, n = nucleus, py = pyrenoid, rcw = ruptured cell wall, sg = starch grain. Scale bar = 500 nm.

In *Xenia elongata* at 28°, viable *Symbiondinium* cells exhibited a smooth coccoid shape and distinct cellular components, such as multi-lobed, elongated chloroplasts and lamellae. They also had a spherical nucleus with well-developed chromosomes, chloroplasts, and pyrenoid bodies (Fig. 4a). After 12 hrs at 30°C, fragmented chloroplasts and aggregated organelles appeared – an indication of stress and degenerative cell death (Fig. 4b). Within 6 hrs at 32°C, zooxanthellar cells showed symptoms of late-stage apoptosis (Fig. 4c) including organelle fragmentation, development of apoptotic bodies, and cell disintegration. Necrosis was also observed, characterized by complete cell organelle rupture (Fig. 4d).

### Temporal Changes in the Morphology of Symbiodinium Zooxanthellae in Hospite - Sarcophyton Ehrenbergi at 34°C

**Figure 5:**
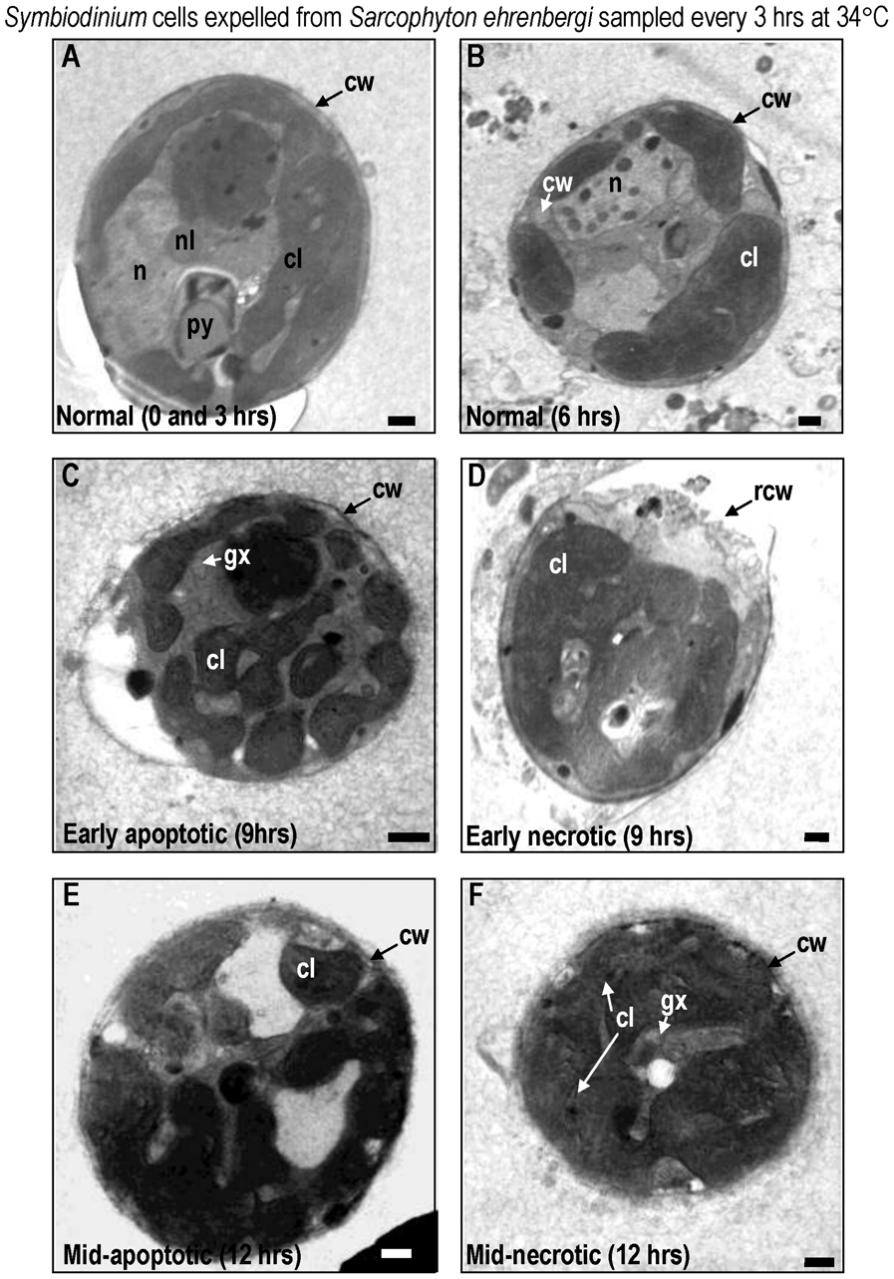
TEM images of *Symbiodinium* cells lost from *Sarcophyton ehrenbergi* through a 12 h period at 34°C, in sequence. Images represent samples taken every 3 h. Note the early onset of specific stages of apoptosis (C) and necrosis (D) within the symbiont cells within 9 h. Within 12 h, mid- to late- apoptotic (E) and necrotic (F) stages represent non-reversible phases. Symbols: chr = chromosome, cl = chloroplast, gx = glyoxysome, m = mitochondria, n = nucleus, py = pyrenoid, sg = starch grain. Approximately 100 zooxanthellar (n = 100) cells were randomly examined per experimental temperature at each time interval, to help characterize normal, apoptotic, and necrotic processes. Scale bar = 500 nm.

In *Sarcophyton ehrenbergi,* viable *Symbiodinium* cells lost from the host displayed normal morphological features after 0 and 3 h, even at 32°C (Fig. 5a,b). After 9 h, however, evidence of apoptosis (Fig. 5c) and necrosis (Fig. 5d) emerged at higher temperatures. Exposure to increased temperatures between 9 and 12 h resulted in the disintegration of cell organelles and their fusion to form one large body, particularly in apoptotic cells (Fig. 5e). After 12 h, free-floating lamellae from the chloroplasts were observed - a characteristic of necrotic cell death (Fig. 5f; [46]). The cell membrane of *Symbiodinium* also disintegrated and the cytosolic contents were released.

### Apopotic and Necrotic Symptoms in in Hospite Cells, Determined by Fluorescent Microscopy (FM)

Viable symbiont cells were ≤12 µm in diameter. Necrotic symbiont cells, on the other hand, reached up to 17 µm in diameter. Cells stained with conjugate Annexin V-*fluor* and observed to be apoptotic were approximately 10 µm in diameter and fluoresced green. At temperatures of 30° to 36°C, frequencies of apoptotic and necrotic *Symbiodinium* cells increased (confirmed with FC; see below) in all three soft coral species - *Sarcophyton ehrenbergi, Sinularia lochmodes*, and *Xenia elongata*. No apoptotic or necrotic coral host cells were observed in either the 28°C control or at ≤34°C. The symptoms did appear, however, at 36°C.

### Apoptotic and Necrotic Symptoms Determined by Flow Cytometry (FC)

The frequency of viable zooxanthellar cells decreased in all three soft coral species as experimental seawater temperatures were increased over the 12 hr period. The number of cells that exhibited apoptosis increased concomitantly.

#### Sarcophyton ehrenbergi *- symbiont* vs. *host cells*

**Figure 6:**
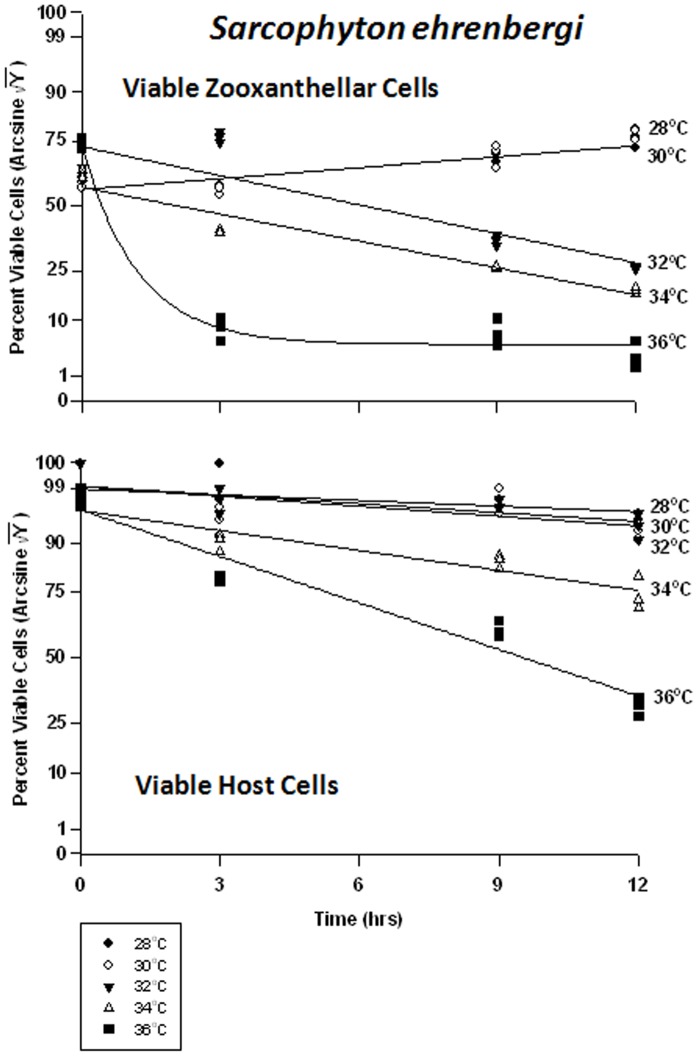
Effects of increasing seawater temperature on percent viable endosymbiotic *Symbiodinium* and host coral *Sarcophyton ehrenbergi* cells. ***In-hospite***, collected over a12 h period. Data shown here relate to corals exposed to 28 to 36°C. • = 28°C; ○ = 30°C;▾ = 32°C; Δ = 34°C; ▪ = 36°C. Data transformed by arcsine for normalization purposes. Each point represents the percentage (%) of different cell types analyzed via TEM. Tissues sampled at 3-hr intervals. (a) **Zooxanthellae:** Significant linear regression in zooxanthellar cells at 28°C and 34°C (p<0.05); Y = 0.843X +49.184, and Y = −2.072+49.688, respectively. Linear trends shown for 30 and 32°C to facilitate comparison (Y = 1.020X +48.679, and Y = −2.243X +59.107, respectively); significant non-linear components at these temperature responses (p<0.05–0.001). Significant non-linear response at 36°C (p<0.001; Y = 13.285+ −46.506*(1−e^[0.838X]^). **Host Cells:** No significant linear or non-linear response at 28°C or 32°C; (slope not significantly different from ‘0′). Significant negative linear response at 34°C (p<0.01; Y = −1.532X +79.187). Significant non-linear components at 30°C and 36°C (p<0.01–0.001); negative linear trends shown into facilitate comparison (Y = −0.762X +84.783, and Y = −3.578X +79.336, respectively).

At normal to slightly increased temperatures (28 to 30°C), the proportion of viable *Symbiodinium* cells *in hospite* in *Sarcophyton ehrenbergi* increased from 55% to approximately 75% within 12 hrs; *i.e.,* no ill effects were observed (Fig. 6a). This may have been due to a natural increase in the zooxanthellae population under non-stress conditions, or may have been due to sampling error. At higher temperatures, however, the frequency of viable cells decreased from 80% to 35% at 32°C, from 40% to 35% at 34°C, and from 80% to 4% at 36°C. The host cells responded quite differently (Fig. 6b). There was no change in cell viability at 28 to 32°C, and viability decreased somewhat within 12 hrs from 90% to 80% at 34°C, and from 80% to 40% at 36°C. Overall, the response of host cells was much lower than zooxanthellar cells to heat stress.

**Figure 7:**
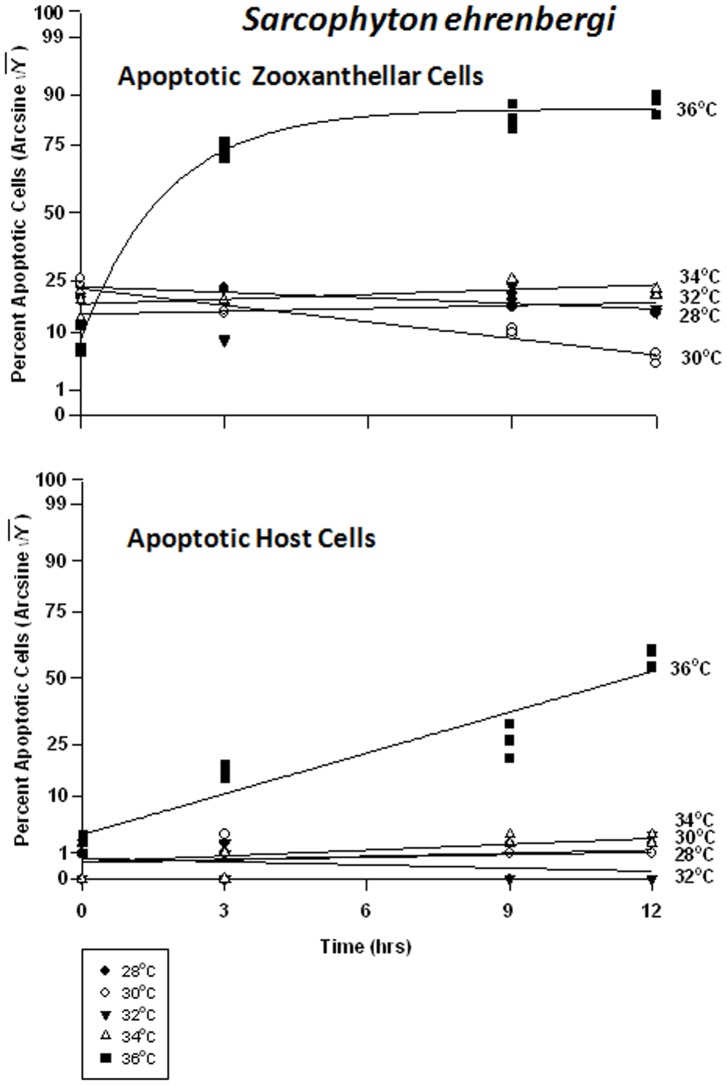
Effects of seawater temperature on percent apoptotic endosymbiotic *Symbiodinium* and host coral *Sarcophyton ehrenbergi* cells. In-hospite, collected over a12 h period. Corals exposed to 28 to 36°C treatments. • = 28°C; ○ = 30°C;▾ = 32°C; Δ = 34°C; ▪ = 36°C. Data transformed by arcsine for normalization purposes. **Zooxanthellae:** Significant negative linear regressions through time at 28°C and 30°C (p<0.05–0.01; Y = −0.413X +28.824, and Y = −1.236X +28.400, respectively). No significant linear at 32°C or 34°C (p>0.05); *i.e.,* regression coefficient not significantly different from “0″. Significant non-linear response at 36°C (p<0.001; Y = 16.608+51.899*[1−−e^(−0.575X)^]). **Host Cells:** No significant linear regression for 28°C, 30°C, 32°C, or 34°C through time (p>0.05); *i.e.,* regression coefficient not significantly different than “0″. Significant non-linear increase at 36°C (p<0.001); linear trend shown to facilitate comparison (Y = 3.068X +9.999).

The frequency of apoptosis in symbiont cells in particular changed only at the highest temperature of 36°C (Fig. 7a). It would appear that the symbionts in *Sarcophyton ehrenbergi* exhibit a steady-state level of apoptosis of between 5 and 30%, even under elevated temperature conditions, until extreme temperatures of 36°C are reached. On the other hand, the host cells have a steady-state level of apoptosis of about 1–5%, which suggests that the host cells, all other things being equal, have a lower turnover rate of cells. Apoptosis frequency decreased significantly through time at 28 and 30°C, from 20% to 18% in the former case, and from 18% to 5% in the latter (Fig. 7a). There was no significant change in apoptosis at 32 and 34°C through time, and the levels of apoptosis were consistent with those at lower temperatures. At 36°C, however, the frequency of apoptosis in symbiont cells rose significantly from 10% to 85%. The host cells, exhibited low levels of apoptosis between 28 and 34°C (Fig. 7b), varying between 1 and 5%; but, at 36°C, the frequency rose dramatically from 5% to 65%.

**Figure 8:**
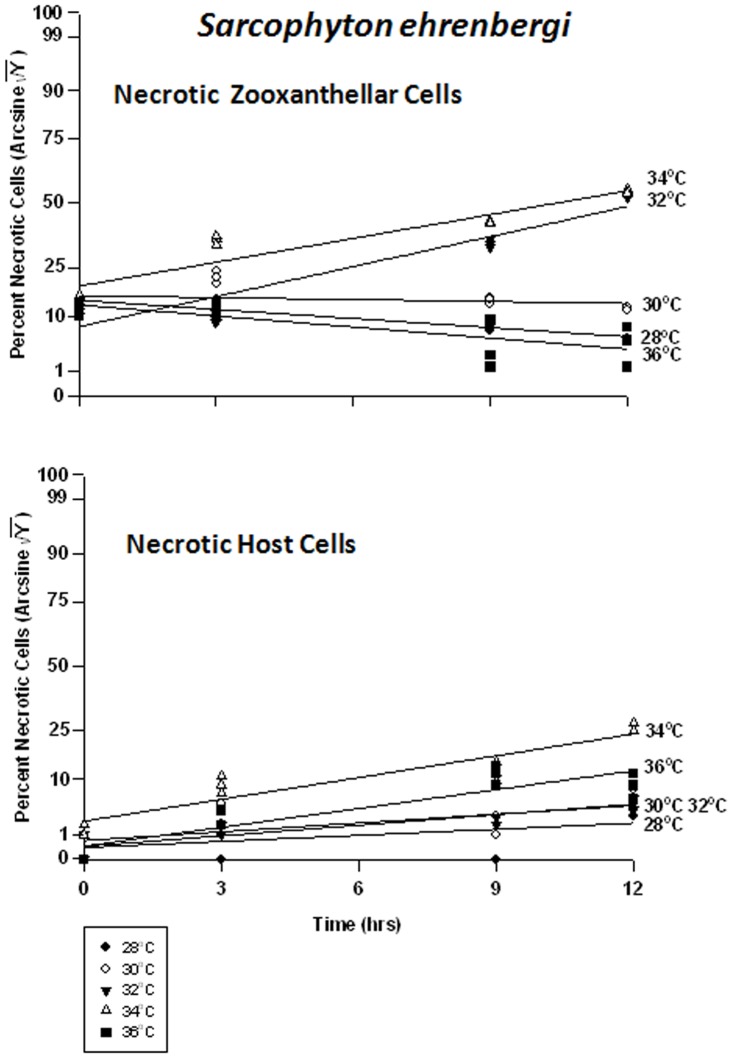
Effects of seawater temperature on percent necrotic endosymbiotic *Symbiodinium* and host coral *Sarcophyton ehrenbergi* cells. In-hospite, collected over a12 h period. Corals exposed to 28 to 36°C treatments. • = 28°C; ○ = 30°C;▾ = 32°C; Δ = 34°C; ▪ = 36°C. Data transformed by arcsine for normalization purposes. **Zooxanthellae:** No significant linear regression at 28°C, 30°C, and 36°C. Significant positive linear regression at 32°C (p<0.05; Y = 2.218X +20.396). Significant non-linear positive response at 34°C (p<0.001); linear trend shown to facilitate comparison (Y = 1.760X +29.396). **Host Cells:** No significant linear regression at 28°C or 30°C (p>0.05); *i.e.,* the regression coefficient is not significantly different from “0”. Significant positive linear regression at 32°C and 34°C (p<0.05; Y = 0.826X +3.017, and Y = 1.701X +8.922, respectively). Positive non-linear response at 36°C (p<0.01); linear trend shown to facilitate comparison (Y = 1.470X +3.080).

At 28 and 30°C, frequency of necrosis in the symbiont cells dropped marginally from 25% to 15%, and 25% to 22%, respectively, over 12 hrs (Fig. 8a). At 32°C, necrosis increased from 15% to 60% and at 34°C, from 30% to 60%. At 36°C, the response of necrotic cells was similar to those at the lower temperatures; however, the frequency of apoptosis inversely rose to ∼65% (see above); we are considering proportions here. Because all proportions must add up to 100%, as one increases, another must decrease; and both apoptosis and necrosis are negative responses to the temperature treatments. The host cells, on the other hand, exhibited increasing but relatively negligible symptoms of necrosis (5% to15%) at 28 through 32°C (Fig. 8b). At 34 and 36°C, necrosis increased from 8% to 25% and 2% to 15%, respectively.

#### Sinularia lochmodes – symbiont vs. host cells

**Figure 9:**
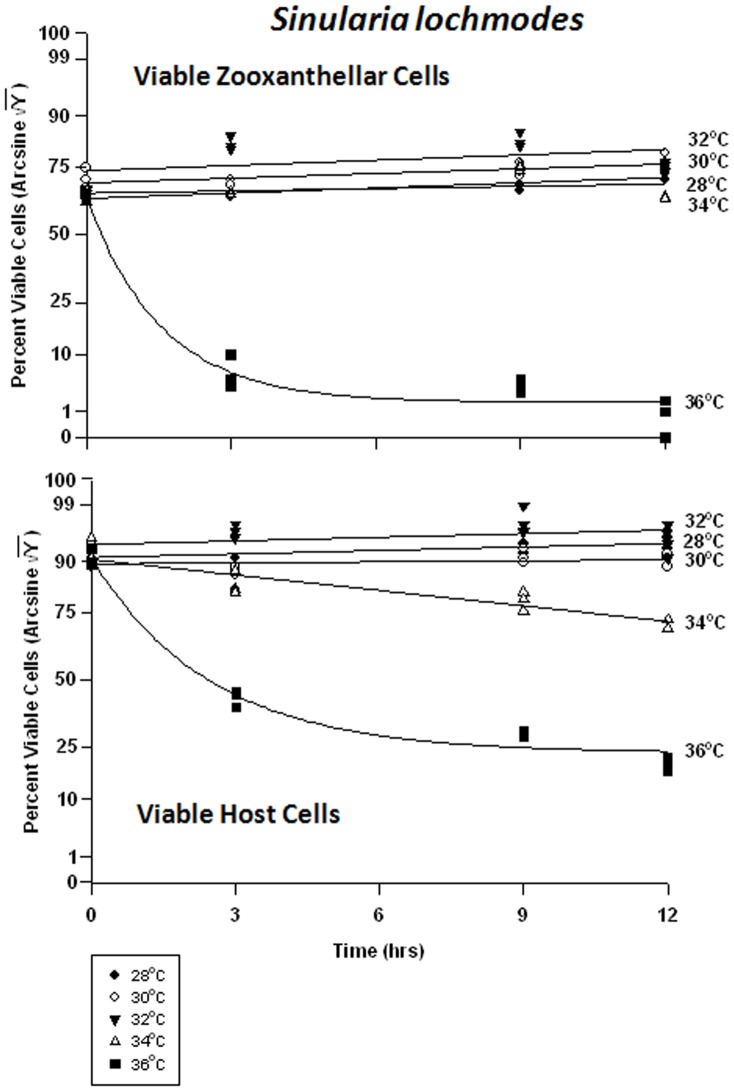
Effects of seawater temperature on percent viable endosymbiotic *Symbiodinium* and host coral *Sinularia lochmodes* cells. *In-hospite* , collected over a 12 h period. Corals exposed to 28 to 36°C treatments. • = 28°C; ○ = 30°C;▾ = 32°C; Δ = 34°C; ▪ = 36°C. Data transformed by arcsine for normalization purposes. **Zooxanthellae:** No significant linear regression at any temperature (p>0.05); *i.e.,* for 28–34°C, regression coefficient not significantly different than “0″. Significant non-linear components at 28°C, 32°C, 34°C, and 36°C (p<0.05–0.001); linear trends shown in the first three of these temperature treatments to facilitate comparison (Y = 0.386X +53.266, Y = 0.393X +59.393, and Y = 0.169X +54.428, respectively). 36°C exponential decay response described by Y = 7.894+45.820*(1−e^[−0.674X]^). **Host Cells:** No significant linear regression for 28°C, 30°C, or 32°C through time (p>0.05); *i.e.,* regression coefficient not significantly different than “0”. Significant negative linear regression at 34°C (p<0.05; Y = −1.149X +72.204). Highly significant non-linear negative response at 36°C (p<0.001; Y = 29.058+43.441*[1-e^(−0.407X)^]).

The symbiont cells in *Sinularia lochmodes* were more resistant to elevated temperatures than those of *Sarchophyton ehrenbergi*. As temperatures increased from 28 to 34°C - known bleaching temperatures, the frequency of viability in *in hospite* cells shifted from 65% to 85% and 75% to 80%, respectively (Fig. 9a). Only at 36°C was cell viability clearly affected, dropping to 10% after 3 hr, and 1% after 12 hrs. The host cells, on the other hand, exhibited little change in viability at 28 through 32°C (Fig. 9b). At 34°C, cell viability frequency dropped from 85% to 75%, and at 36°C, from 70% to 25%.

**Figure 10:**
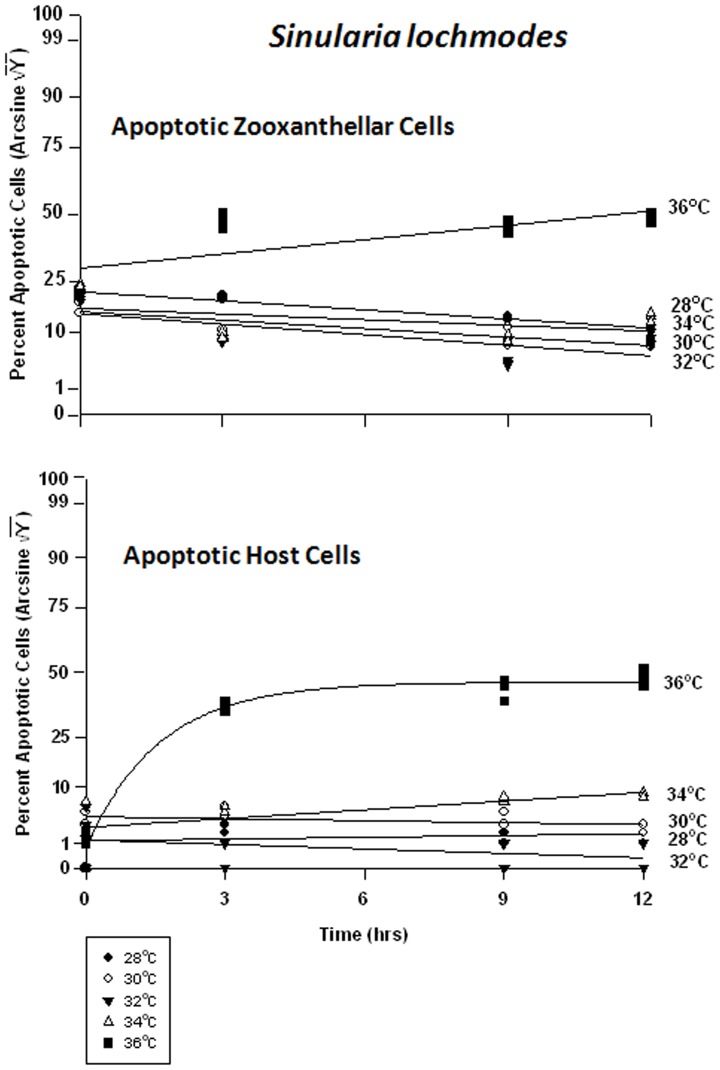
Effects of seawater temperature on percent apoptotic endosymbiotic *Symbiodinium* and host coral *Sinularia lochmodes* cells. ***In-hospite***, collected over a 12 h period. Corals exposed to 28 to 36°C treatments. • = 28°C; ○ = 30°C;▾ = 32°C; Δ = 34°C; ▪ = 36°C. Data transformed by arcsine for normalization purposes. **Zooxanthellae:** Significant negative linear regression at 28°C (p<0.05; Y = −0.682X +27.571). Linear trends shown for 30, 32, 34, and 36°C to facilitate comparison (Y = −0.640X +23.105; Y = −0.796X +22.659; Y = −0.427X +23.837; and Y = 1.077X +32.816, respectively). Significant non-linear responses at 30–34°C (p<0.05–0.001). Significant non-linear trend at 36°C as well (p<0.001). **Host Cells:** No significant linear or non-linear regression at 28–34°C (p>0.05); linear trends shown to facilitate comparison. Significant negative non-linear response at 36°C (p<0.001; Y = 4.630+38.271*[1−e^(−0.634X)^]).

There were highly significant responses in the apoptosis frequency in both the zooxanthellae and the host cells between temperatures and between times, but not between tanks. Apoptosis in the symbionts of *Sinularia lochmodes* decreased through time, but only marginally (Fig. 10a). At 28 through 34°C, apoptosis frequency ranged between 5% and 30%. At 36°C, apoptosis reached levels of 50% in as little as 3 hrs and remained relatively constant through time. The host cells exhibited relatively low and constant apoptosis levels between 28 to 34°C, varying between 0% and 8% (Fig. 10b). At 36°C, the frequency of apoptosis in host cells was higher, increasing from 1% to 50% through time.

**Figure 11:**
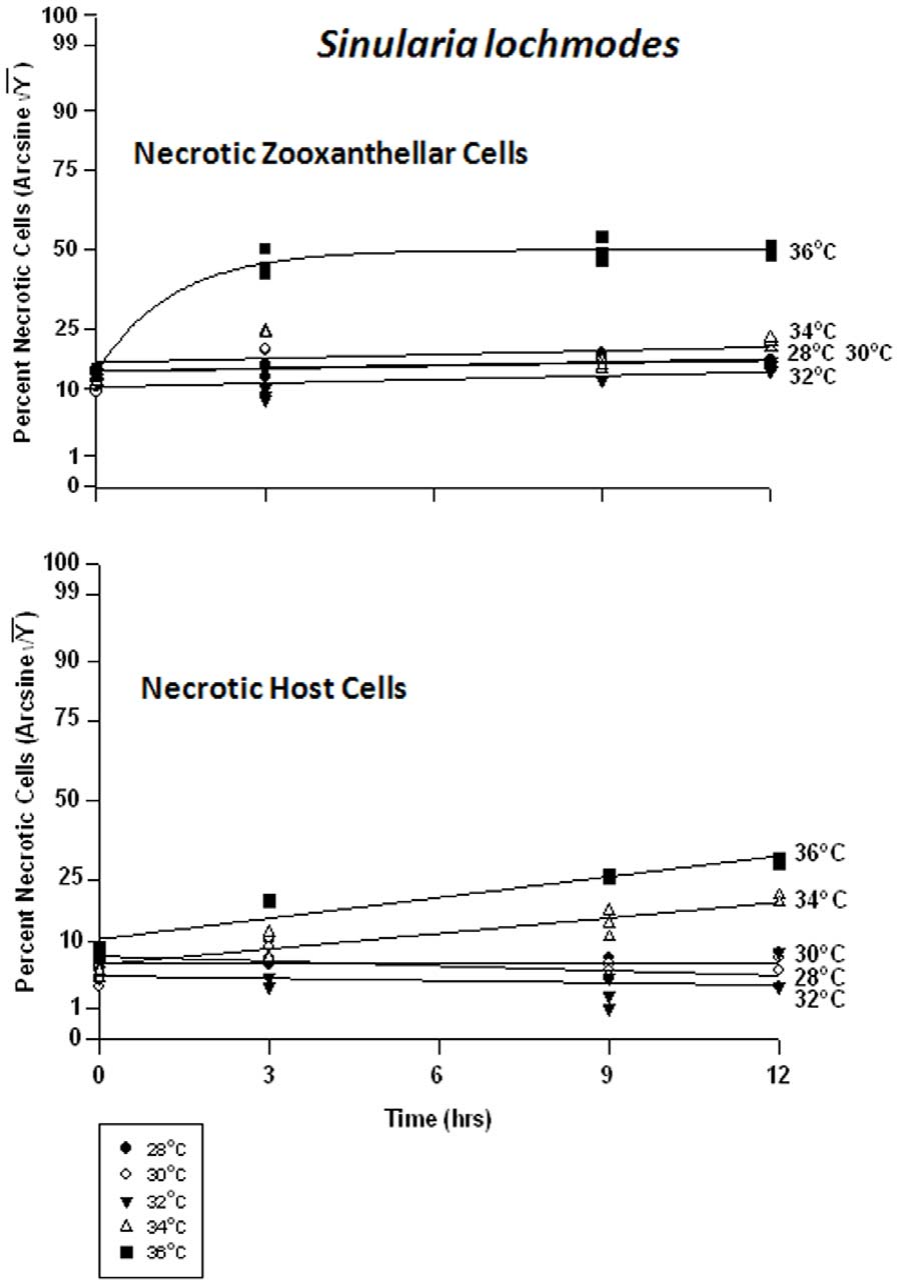
Effects of seawater temperature on percent necrotic endosymbiotic *Symbiodinium* and host coral *Sinularia lochmodes* cells. *In-hospite* , collected over a12 h period. Corals exposed to 28 to 36°C treatments. • = 28°C; ○ = 30°C;▾ = 32°C; Δ = 34°C; ▪ = 36°C. Data transformed by arcsine for normalization purposes. **Zooxanthellae:** No significant linear regressions at 28–34°C (p>0.05); *i.e.,* linear regression coefficients were not significantly different from “0″. Significant deviations from linearity and non-linear response at 36°C (p<0.001; Y = 21.964+23.434*[1−e^(−0745X)^]). **Host Cells:** No significant linear regressions at 28–32°C (p>0.05); *i.e.,* regression coefficients were not significantly different from “0”. Significant positive linear regressions at 34–36°C (p<0.05; Y = 0.979X +14.152, and Y = 1.214X +18.952, respectively).

There was a significant difference in the frequency of necrosis in the zooxanthellae between temperatures, but not between times. There was also a significant difference between tanks and all temperatures. The frequency of necrosis in host cells between temperatures and between times also varied significantly, but not between tanks. Responses of host cells were significantly different under all temperatures. The pattern of necrotic frequency in the symbionts of *Sinularia lochmodes* was generally similar to that of apoptosis (Fig. 11a). The *Sinularia* host cells, however, exhibited only marginal necrosis at 28 through 32°C (Fig. 11b). At 34°C, necrotic frequency increased from 7% to 20%, and at 36°C, from 15% to 35%.

#### Xenia elongata – symbiont vs. host cells

**Figure 12:**
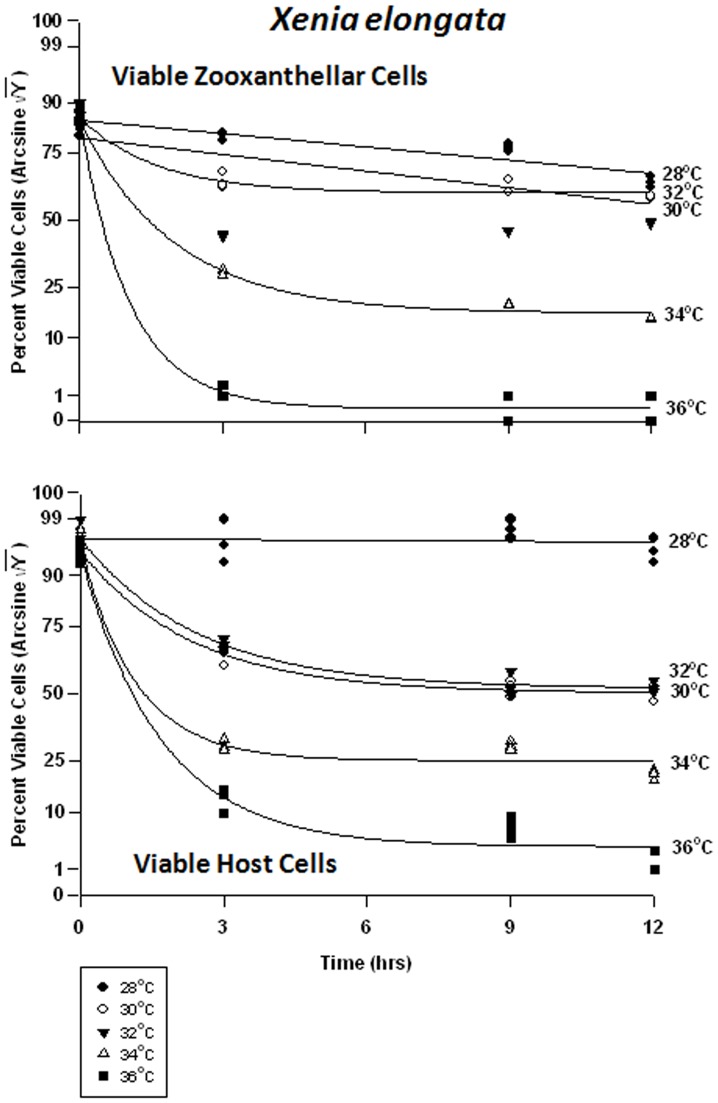
Effects of seawater temperature on percent viable endosymbiotic *Symbiodinium* and host coral *Xenia elongata* cells. In-hospite, collected over a12 h period. Corals exposed to 28 to 36°C treatments. • = 28°C; ○ = 30°C;▾ = 32°C; Δ = 34°C; ▪ = 36°C. Data transformed by arcsine for normalization purposes. **Zooxanthellae:** No significant linear regression at 28–30°C (p>0.05); *i.e.,* regression coefficient not significantly different from “0″. Significant non-linear responses at 32–36°C (p<0.001; Y = 24.315+43.420*(1-e^[−0.507X]^); Y = 2.859+65.500*(1−e^[−0.959X]^); and Y = 45.335+31.910*(1−e^[−0.434X]^), respectively). **Host Cells:** No significant linear regression at 28°C (p>0.05); *i.e.,* linear regression coefficient not significantly different than “0″. Significant non-linear responses at 30, 32, 34, and 36°C (p<0.001; Y = 46.369+33.664*(1−e^[−0.411X]^); Y = 30.191+48.525*(1−e^[−0.879X]^); Y = 10.899+65.806*(1−e^[−0.597X]^), and Y = 51.409+16.910*(1−e^[−0.625X]^), respectively).

In the host cells of *Xenia elongata*, there was a significant difference in viability between temperatures at all times. Analyses revealed that *Xenia elongata* was the most sensitive of the soft corals tested, to temperature. Symbiont cell viability exhibited a graded response to temperature (Fig. 12a), with the highest viability frequency being observed at 28°C, falling from 80% to 70% within 12 hrs. Viability frequency continued to decrease as temperature increased, becoming most severe at 36°C, approaching 0% within 3 hrs. The response of *Xenia* host cell viability was variable, exhibiting higher sensitivity to heat stress than the other two soft coral species, particularly *Sinularia lochmodes* (Fig. 12b). At 28°C, *Xenia* host cell viability remained at ∼95%, but at 30 and 32°C, that viability dropped from ∼75% to ∼55% within 9 hrs. At 34°C, viability was generally lower and dropped from 75% to 25%. Exposure of *Xenia* host cells to 36°C caused the most severe response, with viability dropping from 75% to ∼2% after 12 hrs.

**Figure 13:**
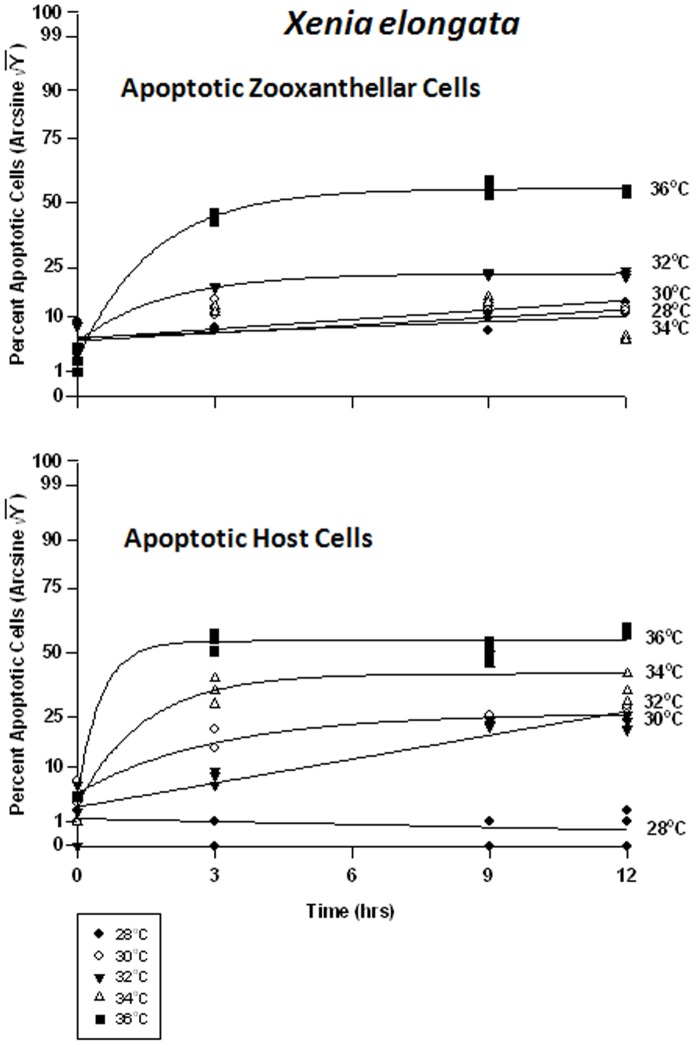
Effects of seawater temperature on percent apoptotic endosymbiotic *Symbiodinium* and host coral *Xenia elongata* cells. In-hospite, collected over a12 h period. Corals exposed to 28 to 36°C treatments. Data transformed by arcsine for normalization purposes. • = 28°C; ○ = 30°C;▾ = 32°C; Δ = 34°C; ▪ = 36°C. **Zooxanthellae:** Significant positive linear regression at 28°C (p<0.05; Y = 0.601X +13.028). Significant non-linear increase in response at 30°C and 34°C (p<0.001); linear trend shown to facilitate comparison (Y = 0.748X +13.474, and Y = 0.415X +13.746, respectively). Significant non-linear responses at 32°C and 36°C (p<0.001; Y = 12.646+16.134*(1−e^[−0.530X]^), and Y = 8.464X +40.198*e^[−0.608]^, respectively). **Host Cells:** No significant linear regression at 28°C (p>0.05); *i.e.,* regression coefficient not significantly different than “0″. Significant non-linear responses at 30°C, 34°C, and 36°C (p<0.001; Y = 9.592+30.720*e^[−0.686X]^, Y = 11.537+36.441*e^[−1.955X]^, and Y = 12.327+18.706*e^[−0.331X]^, respectively). Significant positive linear response at 32°C (p<0.05; Y = 1.864X +9.113).

In the host cells, there were significant differences between temperatures at all times. At 28°, 30, and 34°C, apoptotic frequencies of *Xenia*’s symbiont cells increased from about 1% to 15%, respectively (Fig. 13a). At 32°C, it reached a peak of 23% after only 3 hrs, remaining relatively constant thereafter. The highest apoptotic frequencies were observed at 36°C, increasing from 4% to approximately ∼5% in 3 hrs. *Xenia*’s host coral cells exhibited low, relatively constant levels of apoptosis at about 1–2% at 28°C (Fig. 13b). At temperatures of 30 and 32°C, the frequency increased from 5% to ∼30%. At 34°C, apoptosis frequency rose from 7% to 45% after 3 hrs, remaining relatively constant after that. At 36°C, apoptosis increased from 8% to 55% in as little as 3 hrs.

**Figure 14:**
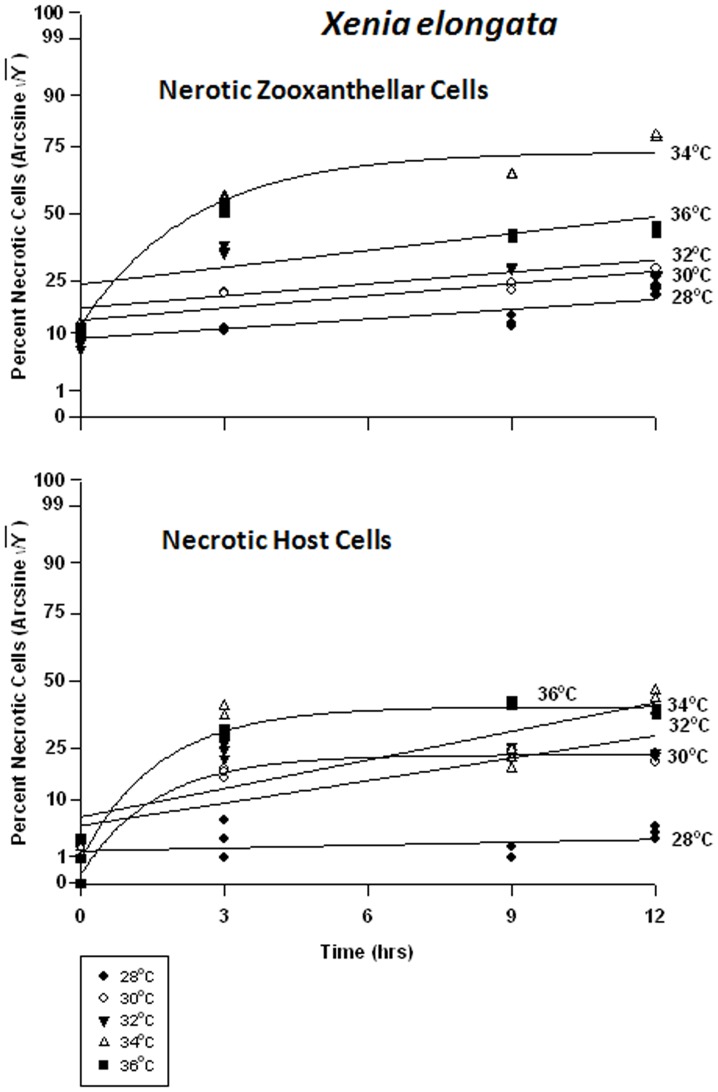
Effects of seawater temperature on percent necrotic endosymbiotic *Symbiodinium* and host coral *Xenia elongata* cells. *In-hospite* , collected over a12 h period. Corals exposed to 28 to 36°C treatments. • = 28°C; ○ = 30°C;▾ = 32°C; Δ = 34°C; ▪ = 36°C. Data transformed by arcsine for normalization purposes. **Zooxanthellae:** Positive non-linear responses in all cases of 28–36°C (p<0.001); linear trends shown for 28, 30, 32 and 36°C cases to facilitate comparison (Y = 0.722X +17.659, Y = 0.905+21.732, Y = 0.889X +24.473, and Y = 1.258X +29.726, respectively). Non-linear response shown for 34°C (Y = 20.375+38.923*(1−e^[−0.427X]^). **Host Cells:** No significant linear regression at 28°C (p>0.05); *i.e.,* regression coefficient not significantly different than “0”. Significant positive non-linear responses at 30–36°C (p<0.001). Relationships at 30°C and 36°C described as Y = 1.911+26.890*(1−e^[−0.640X]^) and Y = 5.232+34.343*(1−e^[−0.612X]^), respectively. Linear trends shown in cases of 32 and 34°C to facilitate comparison (Y = 1.678X +12.990 and Y = 2.132X +14.895, respectively).

Necrotic frequencies were also significantly different in both cell types between temperatures at all times. In *Xenia*’s symbionts, necrotic frequency ranged from 10% to 30% at 28–32°C (Fig. 14a). After 3 hrs at 34°C, necrosis had already reached 55% from 12%. At 36°C, necrosis frequency similarly reached ∼50% in the same amount of time. In the host cells, necrosis remained at about 2% for the 12 hr duration of the experiment. At temperatures of 30°C through 36°C, peak temperatures were reached after only 3 hrs, and these temperature sensitivities were sequentially higher with increasing temperature. These temperatures ranged from 20% to 40%, from 3%.

## Discussion

An understanding of the stability between coral host and its symbiotic zooxanthellae is germane to understanding homeostasis in this dynamic relationship [113]–[118]. Comparing degrees of sensitivity in host *vs.* endosymbiont cells *via* microscopic examination can provide a better understanding of early cytological mechanisms that initiate breakdown in the relationship between the two. Using TEM we were observed *Symbiodinium* from both *Sarcophyton ehrenbergi* and *Sinularia lochmodes* at these temperatures*;* their cell organelles exhibited no degenerative effects. The TEMs also revealed, however, that *Symbiodinium* started exhibiting standard diagnostic symptoms of apoptosis and necrosis at higher temperatures, starting at 32°C and exhibiting acute morphological degenerative changes above this.

There was clear evidence that *Symbiodinium* lost from *Xenia elongata* were more heat-sensitive than those lost from the other two octocoral holobionts and the three other scleractinian coral species examined earlier [46]. Because of this high heat sensitivity in *Xenia*, it is possible that populations of this species could become affected before major bleaching events are noticed, making it a biological indicator species. That is, the physiological responses of this species might serve as a good environmental diagnostic for stress during higher SSTs. Transmission electron microscopy analysis revealed that symptoms of apoptosis and necrosis were evident at seawater temperatures as low as 30°C. The fact that cell organelle structure and integrity was compromised at temperatures of 32°C indicates that this soft coral and its endosymbiotic zooxanthellae should not be expected to survive sustained periods of above-average seawater temperatures.

Many earlier studies focus on heat stress occurring over a temporal scale of weeks, including degree heating weeks (DHW [79]). This study reveals that expelled *Symbiodinium* cells in some corals, such as *Sarcohphyton ehrenbergi*, begin exhibiting signs of potentially reversible PCD after 9 hrs, and signs of irreversible PCD after 12 hrs. Thus, predicting coral bleaching or cell damage on the basis of heat exposure for on the order of hours or days, *vs.* weeks, may be possible. The endosymbionts are susceptible to elevated seawater temperature and exhibit signs of dying or mortality before the end of the first day of exposure. Complete manifestation of the bleaching process does not appear until some time after this, indicating that additional time is required for the expulsion of most of the damaged zooxanthellae. Thus, physiological responses such as those revealed here may serve as an excellent short-term diagnostic of oncoming bleaching. These responses may vary seasonally, but such is not yet known.

Symbiont cells *in hospite* were equally sensitive to elevated temperatures as those cells expelled from *Sarcophyton ehrenbergi* over the same time period. This was evident through parallel changes in frequency of viability. Both apoptosis and necrosis were strong indicators of heat stress. The frequencies of these cell states were much higher in the *in hospite* zooxanthellae than in the host coral cells, the latter exhibiting few signs of apoptosis, even at temperatures of 34°C. Again, this indicates that these symbiotic zooxanthellae are dying very soon after exposure to elevated seawater temperatures. The host animal cells were clearly more tolerant to all experimental temperatures, indicating better thermal adaptation. This pattern is very similar to that observed for both the endosymbiotic cells and the host cells in scleractinian corals studied earlier [42]–[47], [50].

Data from *Sinularia lochmodes* showed that responses of both zooxanthellar and host cells were different from the other species, and thus indicated a level of inter-specific variability not previously suspected. The *in hospite* zooxanthellae of *Sarcophyton ehrenbergi* were more sensitive to temperature than those of *Sinularia* until the latter reached the extreme temperature of 36°C. On the other hand, the responses of the *Sinularia* host cells were similar in the two species. These two soft corals are from different genera and different families, yet their cells exhibited the same thermal tolerances. It is clear that, despite the fact that both species apparently possess the same clade of zooxanthellae – Clade C, the zooxanthellar sensitivities within these hosts are significantly different. This suggests two possible explanatory hypotheses. Firstly, *Sinularia lochmodes* may possess a sub-clade of C which is much more temperature tolerant than the sub-clade in *Sarchophyton ehrenbergi*. Secondly, *Sinularia lochmodes* is tightly packed with calcite spicules which, along with integument of the colony, may better insulate the zooxanthellae from short-term temperature perturbations (≤12 hrs) such as those imposed in this experiment.

Clearly, the host cells of *Xenia* were more sensitive to increasing temperatures than the other two genera. There are two possible explanations for this. Firstly, both *Sarcophyton ehrenbergi* and *Sinularia lochmodes* possess a stiff, leathery, protective integument on the exterior of the polypary. This is supported by densely packed calcium carbonate sclerites within the coenenchyme. *Xenia elongata*, on the other hand, is very lightly calcified, bearing sparse calcite spicules [119]. It is likely that it is not as well insulated from seawater temperature and thus might be expected to absorb heat at a higher rate than the other two species. In addition, it is unlikely that direct absorption, reflection, and refraction of light [120], [121] and associated heating is having any significant effect on colony temperature in these species, since they are completely immersed in seawater which has a high thermal capacity. In addition, our experiments were short-term (≤12 hrs) *vs.* other slightly longer-term experiments (48 hrs [25], [42]), and the latter may have permitted more time for heat absorption by all test organisms.

The zooxanthellae of the alcyonacean corals examined here vary in temperature sensitivity in comparison to those of scleractinian corals studied earlier [25], [42], [46], [88]. Some octocorals such as *Sinularia lochmodes* may have a higher probability of survival in major bleaching events than some scleractinian corals, while *Xenia* would probably experience mortality earlier. Thus, *S. lochomodes* would not represent a good indicator of environmental temperature perturbation while *X. elongata* clearly would.

The presence of apoptosis and necrosis in both the host and symbiont cells of these soft corals, early after exposure to higher temperatures, would cause cells to lyse and release necrotic enzymes, such as lysozymes. This would further damage healthy cells. Zooxanthellae normally resist digestion by the host lysosomes when alive [122]. Further work is needed using TEM and specific stains to determine if the degenerative and apoptotic *in hospite* zooxanthellae are being digested by host lysosomes under thermal stress [80], [87], [123]. This would also amplify effects of cell damage and death in a positively exponential or logarithmic manner, rapidly driving the numbers of affected cells up.

The question arises as to why there is a higher proportion of apoptotic and necrotic cells expelled from the soft corals than there are *in hospite*. The most parsimonious explanation of this difference is that the host coral is well-adapted to sensing cells which are decreasing in functionality or emitting molecules signaling apoptosis or necrosis. After that, it would appear that the damaged cells are transported and expelled [46]. Another aspect of this is that, when the cells are *in hospite*, they are insulated or protected from the changing environment outside the coral. When expelled, they are essentially naked and completely exposed to these elevated temperatures, increasing the rate of cell death.

The fraction of viable *Symbiodinium* cells lost from our experimental soft corals at a normal temperature of 28°C was relatively high. This is an indication that the release of these cells is part of the natural turnover of zooxanthellae – part of their natural population dynamics [25], [124]. That is, it is a background process. An increase in temperature to 32°C, however, resulted in the soft corals exhibiting a lower rate of loss of viable *Symbiodinium* cells, primarily because those cells being released were no longer viable but were apoptotic or necrotic.

### Implications for Zooxanthellar/Host Coral Relationships

The higher sensitivity of zooxanthellae in some alcyonacean soft corals to thermal stress in comparison to their host cells is the same phenomenon as was observed in earlier experiments with scleractinian corals. The inter-specific differences, however, are more pronounced in the alcyonaceans than in the scleractinians. The fact that this high sensitivity to increased temperature in the zooxanthellae of several soft corals contrasts with the generally low sensitivity of the host cells indicates adaptation or exaptation to high seawater temperatures by the host corals. This further suggests that the zooxanthellae may be generally setting the physiological temperature limits for these symbiotic cnidarians in this region - not the host cnidarians. The zooxanthellae here appear to be doing the majority of the adaptation in these species of octocorals [50].

This adaptation (or exaptation) is clearly represented in both of these two cnidarian groups – the Octocorallia-Alcyonacea and the Anthozoa-Scleractinia within this study region, albeit with species-specific variation. There are, however, other groups of Cnidaria that possess zooxanthellae. These include the Anthozoa, Actinaria *(e.g., Aiptasia pallida*) and the Scyphozoa (*e.g., Cassiopeia xamachana*). *A. pallida* is known to bleach at high temperatures [125]. This raises the question as to whether the zooxanthellar-host relationship described above extends beyond the Octocorallia and Anthozoa to other classes within the Cnidaria. Further, it is also possible that, over evolutionary time, the obligate symbiotic relationship between the host and zooxanthellae may be weakened if the requirement of calcification were to diminish. This could occur under increased ocean acidification conditions.

Zooxanthellae also occur in hundreds of other marine invertebrates, some of which secrete calcium carbonate skeletons. For example, they occur in the Platyhelminthes, *i.e.* flatworms (e.g., *Amphiscolops lanerhansi* [126]) and the Mollusca, Bivalvia *(e.g., Tridacna gigas).* The latter species is known to bleach at high seawater temperatures [127]. The question arises as to whether the host-symbiont relationship illustrated here may also apply to these phyla. We hypothesize that it does. A number of different studies over the years have shown that coral holobionts hosting different and/or multiple *Symbiodium* clades have differences in thermal susceptibilities [102], [103], [128], [129]. Fabricius et al. [130] describe how thermo-tolerant clade D zooxanthellae in two Pulauan scleractinian corals may be contributing to their survival from heat stress and bleaching. In our study, we noted that only clade C zooxanthellae occurred in these corals, yet each holobiont had different temperature susceptibilities. This variability between species could cause a shift in community structure as temperatures increase. Comparatively, Bates et al. [131] investigated survival of Pacific Northeast anemones along an environmental gradient. They described host-species specific differences in zooxanthellae when all other physical parameters were relatively similar.

As mentioned above, the Scleractinia and Octocorallia have been on separate evolutionary paths since the Cretaceous [34], [35]. Since then, these groups have diverged greatly in morphology, physiology, and chemical composition [132]. There are some parallels in the two regarding the symbiotic relationship between the zooxanthellae and their hosts with respect to differential temperature sensitivity, and that sensitivity varies greatly between species.

## Materials and Methods

### Target Species

*Sarcophyton ehrenbergi* (von Marenzeller 1886), *Sinularia lochmodes* (Kolonko 1926), and *Xenia elongata* (Xeniidae; Dana 1846; Sprung and Delbeek, 1997) were collected at 7–10 m depths from fringing reefs on Barren Island (23°10′S, 151°55′E) and Outer Rocks (23°4.3′S, 151°57.2′E), on the Great Barrier Reef, off the east coast of central Queensland, Australia. Thus, our results here may be most relevant to soft corals occurring in shallow water (≤10 m), depths particularly susceptible to temperature increases. All corals were maintained in a 200-L holding tank containing 1 µm-filtered seawater (FSW) subjected to a 12 h light:12 h dark –light regimen (150 µmol quanta m^–2^ s^–1^) for 96 h prior to being used in bleaching experiments. *Sarcophyton ehrenbergi* was used because it is known to be less sensitive to heat stress than other species in the region [47], [89], [90], [95], [96], [129], [130]. *Xenia* was chosen because it has been shown to be more heat-sensitive than *Acropora hyacinthus* (Dana 1846), a very sensitive scleractinian coral, to even small increases in seawater temperature [47]. Molecular analysis (18S rDNA) indicated that all of our experimental species harbored only clade C *Symbiodinium*.

Because the experimental colonies were drawn from shallow water, interpretation of results is limited to these depths. It is possible that shallower populations may be exposed to higher/more variable SSTs, and the capacity for adaptation may be more evident in this environment. This is opposed to corals from deeper, less variable depths which have a diminished need to adapt to increased SSTs.

### Experimental Design

Details of the techniques used to isolate and examine *Symbiodinium* cells expelled from corals *via* a flow-through design may be found in Strychar et al. [47]. Data presented here are derived from the same set of experiments, but we have shifted the focus for analysis to *in hospite* host and *Symbiodinum* cells, as opposed to expelled cells (see [47]). Data on expelled cells have been covered in earlier publications. Results from this study will be discussed in the context of earlier ones. Collection of the corals used here was performed under Permit #G99/293 issued by the Great Barrier Reef Marine Park Authority (GBRMPA), Townsville, Qld., Australia.

The experiment followed a randomized block design [131], testing effects of different experimental temperature regimes on these soft corals over a 12 h period. Details of the experimental design are presented in Strychar et al. [46], [47]. We will summarize those techniques here. Ten incubation containers were used for each run. Temperatures within chambers were also randomized for each run. Corals were incubated at average temperatures of 28°, 30°, 32°, 34°, and 36°C. The 28°C temperature acted as a control and was similar to summer mean seawater temperature in the region [46], [47]. Two replicate incubation containers were used for each temperature at each run. This number of containers was used because each set-up was extensive and costly to construct and maintain. A sample size of ten replicates (n = 10) for each coral at each temperature was used. This equates to 30 coral samples used per temperature (i.e. ten of each coral type), 150 coral samples used in total (i.e. 50 samples of each type). The control and experimental temperatures were chosen based upon mean monthly averages available from Integrated Global Ocean Services System (IGOSS; [132]). The control temperature represented the yearly peak average for the region.

Experimental temperatures chosen for this experiment were based upon actual sea surface temperatures near these reefs from 1981 to 2000; temperatures have been predicted through 2100 [132]. The model predicting those temperatures was an Auto-Regressive, Integrated, Moving-Average model (ARIMA), and we used Systat V 9.0. Most severe bleaching events have occurred since 1981 [133], [134]. The model predicted that mean temperatures between 1981 and 2100 would be a yearly average of 26.97°C and a yearly peak average of 31.85°C. Podestá and Glynn [135] and Sandeman [136] observed noticeable symptoms of stress within corals at temperatures higher than 30°C. We also tested the extreme temperatures of 34° and 36°C because Hoegh-Guldberg [137] and Goreau *et al*. [138] have predicted that global warming will produce temperatures similar to these high temperatures between 2001 and 2100. Although temperatures of 36°C have not been observed on the Great Barrier Reef, such high temperatures are common in the Persian Gulf (>33°C [139]), and are known to fluctuate between 17 and 36°C in the Arabian Gulf [140].

We felt that it was important to use two or three separate techniques to demonstrate the target effects of temperature in this experiment. This was done to verify the results and insure that observed effects were not due to technical anomalies, yielding “false positives”. In this case, the flow cytometry, fluorescent microscopy, and transmission electron microscopy techniques all corroborated the results.

We viewed cells under a fluorescent microscope with a blue light source. We were able to divide cell states into three major categories: viable, characterized by cells stained with HO342 fluorescing blue; apoptotic, characterized by the color green when stained with AV-fluor; and necrotic, characterized by a red color when stained with AV-fluor [47].

At the end of each run, the incubation containers were washed in phosphate-free detergent and then rinsed, first with 70% (v/v) ethanol and then filtered seawater (FSW). This rinsing regime was used between runs to help remove any algae and/or metabolites that may have attached to the inside of the incubation container [46], [47]. A sample size of ten replicates (n_i_ = 10) was used for each coral at each temperature. Replicates consisted of independent coral colonies from a given species with similar wet weights.

Filtered seawater (FSW) was first pumped through a series of 10 and 1 µm filters and deposited into four aerated 250 l header tanks (HT), with the water in each being mixed *via* submersible pumps. At a constant flushing rate of 20 ml min^−1^, a peristaltic pump forced the FSW through 1 µm Millipore© depth filters (to remove algal growth), into 2 L incubation chambers. The FSW was mixed within each chamber with an octagon wedge-shaped magnetic stir bar to prevent settlement of *Symbiodinium* lost from the corals. We are confident that the use of a wedge-shaped stir bar plus a high flushing rate (1.2 L hr^−1^, or 3.6 L every 3 h) in each of the 2 L incubation chambers, prevented re-sampling of re-circulated cells.

The non-invasive method used to collect expelled *Symbiodinium* cells was heat extraction, plus natural overflow from each plastic holding container. Homogenization of holobiont tissue to collect zooxanthellae was avoided, since it has been shown in other studies that this yields inaccurate mitotic measurements (see [141]). For example, Suharsono and Brown [141] observed significantly different mitotic indices in homogenate *vs.* serial section tissues of corals. Strychar [57], however, noted a greater number of false-positives in live *vs.* dead cells when using water piks, an invasive method, *vs.* a passive method such as the one used here. Here, the 250 ml of FSW was collected from the excurrent outlet of each incubation chamber every 3 h over a 48 h period (see [46], [47]) or every 3 h over a 12 h time period. The shorter period was used because most responses to our treatments were observed within that period. The collected *Symbiodinium* cells were centrifuged at 700×g for 5 min at room temperature (R_t_). A 1 ml aliquot of supernatant was removed, and the remaining supernatant discarded. The pellet containing *Symbiodinium* cells was then re-suspended in that aliquot using phosphate-buffered saline (PBS, pH 7.2; ICN Biomedicals, Irvine, CA, USA) and subdivided into three aliquots. The first sub-sample was analyzed with fluorescent microscopy (FM), the second with flow cytometry (FC), and the third by transmission electron microscopy (TEM).

### Sample Preparation for Transmission Electron Microscopy (TEM)

Approximately 5 mm^2^ of excised coral tissue was taken from each sample colony using a scalpel and pair of fine dissecting forceps. At each sampling interval, separate aliquots of coral host tissue and *Symbiodinium* cells were fixed in 0.2 M phosphate buffered saline containing 3% glutaraldehyde for 24 hrs. Following fixation, both host tissue and *Symbiodinium* cell aliquots were centrifuged (2000×*g*) and washed three-times in 0.1 M Sorensen’s phosphate buffer (pH 7.2; Electron Microscopy Sciences - EMS, Washington, USA) for 10 min. Samples were centrifuged to concentrate the *Symbiodinium* cells, estimated at a concentration of 1–2×10^6^ cells ml^−1^.

In order to examine host cells, samples were decalcified using a solution of sodium citrate (10%) and formic acid (20%), following the methods of Rinkevich and Loya [142] and Banin et al. [143]. After washing three times and removal of the supernatant, the individual host samples, including their zooxanthellae, were embedded in 0.2 ml agar. This allowed us to analyze both symbiont and host cells simultaneously. Expelled symbiont cells were embedded in a similar manner [47]. The agar-embedded samples, once solidified, were post-fixed in 1% osmium tetroxide (EMS) in 0.1 M PBS for 14–24 h at 48°C. The samples were then centrifuged (500×*g*) and the supernatant removed. Samples were dehydrated in a graded series of acetone washes (30, 50, 70, 90 and 3× at 100%) at 30 min intervals. Once dehydrated, the embedded samples were suspended in a series of graded acetone–Spurr’s resin (EMS®) to ensure complete infiltration of the resin into all tissue, and then they were polymerized into a mould by incubation at 60°C for 3 d.

Thin sections of each sample were obtained using an Ultracut T ultra-microtome (Leica Microsystems, Austria). Thin sections were placed on 3 mm copper grids (size 5, 300 mesh) coated with 1.2% weight per volume (w/v) formvar in trichloromethane. They were then stained for 5 min with 2% (w/v) aqueous uranyl acetate followed by 1.5% (w/v) lead citrate for 5 min. In order to examine their ultrastructural characteristics, the samples were observed with a JEOL-1010 or Hitachi 7000 TEM. One hundred host tissue cells and 100 zooxanthellar cells were examined per experimental time interval, per temperature, per host. Characteristics symptomatic of apoptotic, necrotic, and viable cells were logged. Quantitative measurements of organelles were also taken for symbiont cells.

### Sample Preparation for Fluorescent Microscopy (FM)

Fluorescent microscopy was used to confirm the binding and fluorescent properties of flow cytometry (FC) dyes used to detect apoptosis and necrosis. The stain used was a mixture of approximately 4 µl of propidium iodide (PI; 100 mg ml^−1^; Sigma–Aldrich, Castle Hill, NSW, Australia) and 5 µl of Annexin V*fluor* (AV-*fluor*; Bioscientific, Kirawee, NSW, Australia). AV-*fluor* was used to identify cells undergoing apoptosis [47]. The conjugates bind to phosphatidylserine in cells undergoing apoptosis and fluoresce green when excited with a blue light [44]. This mixture was added to aliquots containing 100 µl of *Symbiodinium* cell suspension (1.8×10^5^ cells) and separate aliquots containing host cells. Aliquots containing stain were placed in the dark at room temperature for 20–30 min. Ten ml of cell suspension were then examined under a FM to ensure dye-binding specificity and distinct auto-fluorescence of each conjugate with respect to the host and zooxanthellar cells. A control using only PBS as a medium was used to compare and contrast results obtained from cultures containing stained CS-156 (*Symbiodinium*) cells.

### Sample Preparation for Flow Cytometry (FC)

Aliquots of *in hospite* coral host and *Symbiodinium* cells stained with AV-*fluor* and PI were examined using an EPICS Profile-II cytometer. This functioned to discriminate between apoptotic and necrotic cells, in a manner similar to that described above. Host and *Symbiodinium* cells processed by FC, fluoresce red when stained with PI, green when stained with AV-*fluor*, and do not stain at all if viable [47], [57]. In each study, a minimum of 100,000 events were examined by FC, comparing each group of replicates from each experimental time period (e.g. 3, 9, 12 h), under each temperature treatment (28, 30, 32, 34, and 36°C), assessing their physiological state (*i.e.,* viable, apoptotic, or necrotic). Different colonies and species of soft corals varied in their levels of viable, apoptotic, and necrotic cells. To facilitate comparison between species, relative rather than absolute changes in frequencies were considered. The experiment was replicated for each coral species - ten colonies per treatment.

Controls for comparison with both positive (viable) and negative (apoptotic/necrotic) cells were prepared fresh before each experimental period. Aliquots (100 µL), one for each type of analysis, were centrifuged at 700×g for 5 min at room temperature, to help concentrate the cells (∼ 1–2×10^6^ cells mL^−1^) and remove the slurry. The cells were than re-suspended in 1 mL of F2-enriched filtered seawater (FSW) containing 3 mM CaCl_2_. Each aliquot was than filtered through a 50 µm mesh to reduce cell clumping. To each aliquot, 4 µL of propidium iodide (PI; 100 µg mL^−1^) and 4 µL of AV-fluor were added, and the cells were incubated for 30 min at room temperature in the dark. Prior to FC analysis, 400 µL of F2 enriched (3 mM CaCl_2_) FSW media was added to each aliquot of cells.

### Statistical Analyses

The health of the host and its symbionts under experimental and control conditions were statistically compared using a series of two-way, replicated, balanced, Model I, factorial ANOVAs (α = 0.05 [131]). The two treatments were temperature and time, and the variables were concentrations of viable, apoptotic, and necrotic cells, respectively, from the different host species, respectively. Means were also analyzed using least squares linear regression analysis along with dynamic iterative curve-fitting procedures for non-linear responses. With respect to curve-fitting, the model used for increasing trends was a three-parameter exponential growth to a maximum (asymptote), and, for decreasing trends, a three-parameter exponential decay to a minimum (asymptote) (SigmaPlot 10.0). Proportions were transformed by arcsine (square-root of Y) for normalization purposes. Temperature differences between tanks were tested for using a two-way replicated ANOVA for each of the five experimental temperatures, using species and tanks as primary factors.

To test for the precision of our experimental tank temperatures in regards to our target temperatures, we first calculated grand means for each species at each temperature along with their 95% confidence limits. These were graphed against target temperatures for the experiment. In addition, we performed a Comparison of a Single Observation with the Mean of a Sample test [131] on each of the 150 trials performed in this experiment. In this case, the mean was the average temperature in a run (n_i_ = 36); the single observation tested against that population was the target temperature.

Details of statistical analytical results will be presented in the figure legends and summarized briefly in the text.

## Supporting Information

Table S1 **Actual seawater temperatures for experimental trials.**
(DOC)
