# Supplementary material for: Responses to High Seawater Temperatures in Zooxanthellate Octocorals
Source: PLoS One. 2013 Feb 6;8(2):e54989. doi: 10.1371/journal.pone.0054989 (PMC3566138; doi:10.1371/journal.pone.0054989)
Supplement: Table S1 — Actual seawater temperatures for experimental trials. (DOC) [file pone.0054989.s001.doc]

| **Supplementary Table 1. Actual seawater temperatures for experimental trials.** | | | |  |  |  |  |  |  |  |  |  |
| --- | --- | --- | --- | --- | --- | --- | --- | --- | --- | --- | --- | --- |
|  |  |  | **Actual Experimental Temperatures** | | | | | | | | | |
|  | **Target** |  |  |  |  |  | **Trials** | |  |  |  |  |
| **Species** | **Temp. (^o^C)** |  | **1** | **2** | **3** | **4** | **5** | **6** | **7** | **8** | **9** | **10** |
| ***Sarcophyton*** | **28** | **MEAN** | 28.17 | 28.30 | 28.29 | 28.26 | 28.18 | 28.18 | 28.10 | 28.09 | 28.14 | 28.23 |
| ***ehrenbergi*** |  | **SD= Standard Deviation** | 0.100 | 0.223 | 0.252 | 0.163 | 0.211 | 0.211 | 0.150 | 0.169 | 0.189 | 0.217 |
|  |  | **95% CI = Confidence Interval** | 0.062 | 0.138 | 0.156 | 0.101 | 0.131 | 0.131 | 0.093 | 0.105 | 0.117 | 0.134 |
|  |  | **t_s_** | -1.677 | -1.342 | -1.145 | -1.585 | -0.839 | -0.839 | -0.677 | -0.539 | -0.723 | -1.053 |
|  |  | **Significance** | n.s. | n.s. | n.s. | n.s. | n.s. | n.s. | n.s. | n.s. | n.s. | n.s. |
|  | **30** | **MEAN** | 30.21 | 30.14 | 30.14 | 30.24 | 30.08 | 30.20 | 30.18 | 30.22 | 30.15 | 30.12 |
|  |  | **SD= Standard Deviation** | 0.050 | 0.114 | 0.094 | 0.093 | 0.144 | 0.098 | 0.093 | 0.125 | 0.159 | 0.121 |
|  |  | **95% CI = Confidence Interval** | 0.031 | 0.071 | 0.058 | 0.057 | 0.089 | 0.061 | 0.058 | 0.077 | 0.098 | 0.075 |
|  |  | **t_s_** | -4.164 | -1.213 | -1.469 | -2.590 | -0.588 | -2.024 | -1.994 | -1.763 | -0.928 | -1.025 |
|  |  | **Significance** | *** | n.s. | n.s. | * | n.s. | n.s. | n.s. | n.s. | n.s. | n.s. |
|  | **32** | **MEAN** | 32.00 | 32.01 | 32.02 | 32.06 | 32.21 | 32.05 | 32.10 | 32.12 | 32.05 | 32.05 |
|  |  | **SD= Standard Deviation** | 0.162 | 0.233 | 0.186 | 0.182 | 0.129 | 0.190 | 0.167 | 0.182 | 0.146 | 0.065 |
|  |  | **95% CI = Confidence Interval** | 0.100 | 0.144 | 0.115 | 0.113 | 0.080 | 0.118 | 0.104 | 0.113 | 0.090 | 0.040 |
|  |  | **t_s_** | -0.002 | -0.053 | -0.086 | -0.357 | -1.615 | -0.260 | -0.621 | -0.653 | -0.333 | -0.807 |
|  |  | **Significance** | n.s. | n.s. | n.s. | n.s. | n.s. | n.s. | n.s. | n.s. | n.s. | n.s. |
|  | **34** | **MEAN** | 34.07 | 34.09 | 34.08 | 34.12 | 34.09 | 34.10 | 34.12 | 34.13 | 34.14 | 34.13 |
|  |  | **SD= Standard Deviation** | 0.179 | 0.138 | 0.127 | 0.112 | 0.161 | 0.113 | 0.132 | 0.121 | 0.137 | 0.130 |
|  |  | **95% CI = Confidence Interval** | 0.111 | 0.086 | 0.079 | 0.070 | 0.100 | 0.070 | 0.082 | 0.075 | 0.085 | 0.081 |
|  |  | **t_s_** | -0.378 | -0.646 | -0.613 | -1.096 | -0.554 | -0.942 | -0.934 | -1.111 | -1.065 | -0.987 |
|  |  | **Significance** | n.s. | n.s. | n.s. | n.s. | n.s. | n.s. | n.s. | n.s. | n.s. | n.s. |
|  | **36** | **MEAN** | 36.12 | 36.15 | 36.14 | 36.15 | 36.15 | 36.15 | 36.16 | 36.13 | 36.15 | 36.14 |
|  |  | **SD= Standard Deviation** | 0.085 | 0.074 | 0.079 | 0.087 | 0.085 | 0.076 | 0.069 | 0.087 | 0.067 | 0.080 |
|  |  | **95% CI = Confidence Interval** | 0.053 | 0.046 | 0.049 | 0.054 | 0.052 | 0.047 | 0.043 | 0.054 | 0.042 | 0.049 |
|  |  | **t_s_** | -1.400 | -2.006 | -1.807 | -1.749 | -1.773 | -1.953 | -2.401 | -1.562 | -2.179 | -1.816 |
|  |  | **Significance** | n.s. | n.s. | n.s. | n.s. | n.s. | n.s. | * | n.s. | * | n.s. |
| ***Sinularia*** | **28** | **MEAN** | 28.12 | 28.19 | 28.26 | 28.15 | 28.24 | 28.36 | 28.28 | 28.23 | 28.21 | 28.21 |
| ***lochmodes*** |  | **SD= Standard Deviation** | 0.155 | 0.228 | 0.202 | 0.162 | 0.242 | 0.240 | 0.222 | 0.112 | 0.176 | 0.280 |
|  |  | **CI = Confidence Interval** | 0.096 | 0.141 | 0.125 | 0.100 | 0.150 | 0.149 | 0.138 | 0.069 | 0.109 | 0.173 |
|  |  | **t_s_** | -0.787 | -0.849 | -1.312 | -0.911 | -0.997 | -1.522 | -1.267 | -2.106 | -1.231 | -0.750 |
|  |  | **Significance** | n.s. | n.s. | n.s. | n.s. | n.s. | n.s. | n.s. | * | n.s. | n.s. |
|  | **30** | **MEAN** | 30.12 | 30.15 | 30.20 | 30.27 | 30.12 | 30.23 | 30.11 | 30.24 | 30.01 | 30.03 |
|  |  | **SD= Standard Deviation** | 0.108 | 0.150 | 0.193 | 0.205 | 0.188 | 0.150 | 0.121 | 0.139 | 0.143 | 0.111 |
|  |  | **CI = Confidence Interval** | 0.067 | 0.093 | 0.119 | 0.127 | 0.116 | 0.093 | 0.075 | 0.086 | 0.088 | 0.069 |
|  |  | **t_s_** | -1.137 | -0.994 | -1.040 | -1.327 | -0.655 | -1.537 | -0.884 | -1.740 | -0.059 | -0.303 |
|  |  | **Significance** | n.s. | n.s. | n.s. | n.s. | n.s. | n.s. | n.s. | n.s. | n.s. | n.s. |
|  | **32** | **MEAN** | 32.09 | 32.11 | 32.16 | 32.05 | 32.12 | 32.10 | 32.02 | 32.04 | 32.01 | 32.01 |
|  |  | **SD= Standard Deviation** | 0.147 | 0.135 | 0.158 | 0.121 | 0.144 | 0.156 | 0.156 | 0.115 | 0.096 | 0.096 |
|  |  | **CI = Confidence Interval** | 0.091 | 0.084 | 0.098 | 0.075 | 0.089 | 0.097 | 0.096 | 0.071 | 0.060 | 0.060 |
|  |  | **t_s_** | -0.646 | -0.799 | -1.040 | -0.402 | -0.833 | -0.625 | -0.138 | -0.323 | -0.102 | -0.102 |
|  |  | **Significance** | n.s. | n.s. | n.s. | n.s. | n.s. | n.s. | n.s. | n.s. | n.s. | n.s. |
|  | **34** | **MEAN** | 34.14 | 34.12 | 34.12 | 34.08 | 34.13 | 34.09 | 34.13 | 34.12 | 34.09 | 34.13 |
|  |  | **SD= Standard Deviation** | 0.120 | 0.150 | 0.092 | 0.104 | 0.121 | 0.098 | 0.099 | 0.101 | 0.106 | 0.091 |
|  |  | **CI = Confidence Interval** | 0.074 | 0.093 | 0.057 | 0.064 | 0.075 | 0.061 | 0.061 | 0.063 | 0.065 | 0.056 |
|  |  | **t_s_** | -1.170 | -0.829 | -1.268 | -0.777 | -1.111 | -0.937 | -1.356 | -1.157 | -0.896 | -1.411 |
|  |  | **Significance** | n.s. | n.s. | n.s. | n.s. | n.s. | n.s. | n.s. | n.s. | n.s. | n.s. |
|  | **36** | **MEAN** | 36.11 | 36.12 | 36.14 | 36.11 | 36.14 | 36.12 | 36.13 | 36.10 | 36.11 | 36.10 |
|  |  | **SD= Standard Deviation** | 0.073 | 0.102 | 0.078 | 0.098 | 0.087 | 0.087 | 0.089 | 0.089 | 0.088 | 0.087 |
|  |  | **CI = Confidence Interval** | 0.045 | 0.063 | 0.048 | 0.061 | 0.054 | 0.054 | 0.055 | 0.055 | 0.055 | 0.054 |
|  |  | **t_s_** | -1.553 | -1.151 | -1.797 | -1.183 | -1.634 | -1.451 | -1.441 | -1.112 | -1.298 | -1.203 |
|  |  | **Significance** | n.s. | n.s. | n.s. | n.s. | n.s. | n.s. | n.s. | n.s. | n.s. | n.s. |
| ***Xenia*** | **28** | **MEAN** | 28.22 | 28.20 | 28.41 | 28.20 | 28.16 | 28.28 | 28.23 | 28.17 | 28.16 | 28.15 |
| ***elongata*** |  | **SD= Standard Deviation** | 0.171 | 0.204 | 0.230 | 0.234 | 0.166 | 0.232 | 0.225 | 0.145 | 0.110 | 0.091 |
|  |  | **CI = Confidence Interval** | 0.106 | 0.127 | 0.143 | 0.145 | 0.103 | 0.144 | 0.139 | 0.090 | 0.068 | 0.057 |
|  |  | **t_s_** | -1.305 | -1.012 | -1.789 | -0.846 | -0.961 | -1.205 | -1.056 | -1.153 | -1.434 | -1.666 |
|  |  | **Significance** | n.s. | n.s. | n.s. | n.s. | n.s. | n.s. | n.s. | n.s. | n.s. | n.s. |
|  | **30** | **MEAN** | 30.16 | 30.18 | 30.28 | 30.18 | 30.05 | 30.20 | 30.23 | 30.13 | 30.12 | 30.00 |
|  |  | **SD= Standard Deviation** | 0.145 | 0.166 | 0.182 | 0.148 | 0.158 | 0.165 | 0.167 | 0.153 | 0.168 | 0.178 |
|  |  | **CI = Confidence Interval** | 0.090 | 0.103 | 0.113 | 0.092 | 0.098 | 0.102 | 0.103 | 0.095 | 0.104 | 0.110 |
|  |  | **t_s_** | -1.096 | -1.083 | -1.545 | -1.253 | -0.299 | -1.223 | -1.400 | -0.827 | -0.723 | 0.000 |
|  |  | **Significance** | n.s. | n.s. | n.s. | n.s. | n.s. | n.s. | n.s. | n.s. | n.s. | n.s. |
|  | **32** | **MEAN** | 32.01 | 32.07 | 32.16 | 32.13 | 32.08 | 32.22 | 32.11 | 32.15 | 32.08 | 32.11 |
|  |  | **SD= Standard Deviation** | 0.096 | 0.204 | 0.158 | 0.122 | 0.163 | 0.101 | 0.158 | 0.146 | 0.152 | 0.141 |
|  |  | **CI = Confidence Interval** | 0.060 | 0.126 | 0.098 | 0.076 | 0.101 | 0.063 | 0.098 | 0.091 | 0.094 | 0.087 |
|  |  | **t_s_** | -0.102 | -0.327 | -1.040 | -1.111 | -0.498 | -2.158 | -0.692 | -1.041 | -0.499 | -0.814 |
|  |  | **Significance** | n.s. | n.s. | n.s. | n.s. | n.s. | * | n.s. | n.s. | n.s. | n.s. |
|  | **34** | **MEAN** | 34.10 | 34.12 | 34.08 | 34.11 | 34.12 | 34.13 | 34.14 | 34.11 | 34.12 | 34.13 |
|  |  | **SD= Standard Deviation** | 0.142 | 0.171 | 0.160 | 0.131 | 0.225 | 0.122 | 0.146 | 0.101 | 0.131 | 0.080 |
|  |  | **CI = Confidence Interval** | 0.088 | 0.106 | 0.099 | 0.081 | 0.139 | 0.076 | 0.090 | 0.063 | 0.081 | 0.049 |
|  |  | **t_s_** | -0.717 | -0.726 | -0.522 | -0.852 | -0.556 | -1.057 | -1.006 | -1.100 | -0.896 | -1.682 |
|  |  | **Significance** | n.s. | n.s. | n.s. | n.s. | n.s. | n.s. | n.s. | n.s. | n.s. | n.s. |
|  | **36** | **MEAN** | 36.10 | 36.14 | 36.13 | 36.09 | 36.10 | 36.09 | 36.11 | 36.10 | 36.11 | 36.10 |
|  |  | **SD= Standard Deviation** | 0.084 | 0.083 | 0.094 | 0.084 | 0.089 | 0.086 | 0.077 | 0.097 | 0.082 | 0.106 |
|  |  | **CI = Confidence Interval** | 0.052 | 0.052 | 0.058 | 0.052 | 0.055 | 0.053 | 0.048 | 0.060 | 0.051 | 0.066 |
|  |  | **t_s_** | -1.197 | -1.681 | -1.382 | -1.128 | -1.123 | -1.103 | -1.487 | -1.025 | -1.342 | -0.990 |
|  |  | **Significance** | n.s. | n.s. | n.s. | n.s. | n.s. | n.s. | n.s. | n.s. | n.s. | n.s. |

Supplementary Table 1.

Comparison of mean seawater temperatures in each experimental trial/run with the target treatment temperature. Data analyzed via Comparison of a Single Observation with the Mean of a Sample (147). Mean, standard deviation, 95% confidence interval, t_s_, and level of significance shown for each run in each subset. Data shown for 10 trials within each temperature for each soft coral tested - *Sarcophyton ehrenbergi, Sinularia lochmodes,* and *Xenia elongata.*  This totaled 150 trials. * = p < 0.05, ** = p < 0.01, *** = p < 0.001, n.s. = not significant. Six runs varied significantly from target temperatures: *Sarcophyton ehrenbergi*, 30^o^C, runs #1 and 4; 36^o^C, runs #7 and 9; *Sinularia lochmodes*, 28^o^C, run #8; and *Xenia elongata*, 32^o^C, run #6. Range of differences between the mean experimental temperature and the target in these cases was 0.15 to 0.24^o^C.
